# Characterizing chloroplast genomes and inferring maternal divergence of the *Triticum–Aegilops* complex

**DOI:** 10.1038/s41598-021-94649-9

**Published:** 2021-07-28

**Authors:** Yong-Bi Fu

**Affiliations:** Plant Gene Resources of Canada, Saskatoon Research and Development Centre, Agriculture and Agri-Food Canada, 107 Science Place, Saskatoon, SK S7N 0X2 Canada

**Keywords:** Plant domestication, Plant evolution, Evolutionary genetics

## Abstract

The *Triticum* (wheat)*–Aegilops* (goatgrass) complex has been extensively studied, but the evolutionary history of polyploid wheats has not been fully elucidated. The chloroplast (cp) with maternal inheritance and homoplasy can simplify the sequence-based evolutionary inferences, but informative inferences would require a complete and accurate cp genome sequence. In this study, 16 cp genomes representing five *Aegilops* and 11 *Triticum* species and subspecies were sequenced, assembled and annotated, yielding five novel circular cp genome sequences. Analyzing the assembled cp genomes revealed no marked differences in genome structure and gene arrangement across the assayed species. A polymorphism analysis of 72 published cp genome sequences representing 10 *Aegilops* and 15 *Triticum* species and subspecies detected 1183 SNPs and 1881 SSRs. More than 80% SNPs detected resided on the downstream and upstream gene regions and only 2.78% or less SNPs were predicted to be deleterious. The largest nucleotide diversity was observed in the short single-copy genomic region. Relatively weak selection pressure on cp coding genes was detected. Different phylogenetic analyses confirmed that the maternal divergence of the *Triticum–Aegilops* complex had three deep lineages each representing a diploid species with nuclear A, B, or D genome. Dating the maternal divergence yielded age estimates of divergence that matched well with those reported previously. The divergence between emmer and bread wheats occurred at 8200–11,200 years ago. These findings are useful for further genomic studies, provide insight into cp genome evolvability and allow for better understanding of the maternal divergence of the *Triticum–Aegilops* complex.

## Introduction

Evolutionary history of the *Triticum* (wheat)*–Aegilops* (goatgrass) complex has long been the focus of studies^1–3^, as the *Triticum–Aegilops* complex with 30 species^4^ represents the important wheat group in the tribe of Triticeae with about 330 species^5^. These studies have utilized cytogenetic, genetic, molecular, and phylogenetic tools, and advanced our knowledge about the origin of polyploid wheats^6–8^. Bread wheat has a genome consisting of three closely related subgenomes (AABBDD). It is generally accepted that bread wheat originated from two polyploidization events; first, a tetraploidization some 0.5 million years ago (Mya) following the hybridization between wild *T. urartu* (AA) and an undiscovered species of the *Ae. speltoides* lineage (BB), and second, a hexaploidization some 10,000 years ago as a result of hybridization between a descendant of this original tetraploid hybrid (AABB) and wild diploid *Ae. tauschii* (DD). However, the evolutionary history of polyploid wheats has not been fully elucidated^9–13^. This reflects largely on the complexity of wheat evolutionary history from the divergence of wheat diploid ancestors, through two steps of polyploidization, domestication, and extensive breeding^13,14^. These processes may also involve recurrent intergeneric hybridizations and introgression events as well as incomplete sorting of ancestral polymorphisms^14–17^ and could be lineage-, genome-, and gene copy-specific^18,19^. Thus, many uncertainties remain in the evolutionary inferences of the complex due to the variable nature of DNA sequence data^11,12,17,18,20^. For example, the nuclear gene phylogeny inferred by Marcussen et al.^18^ placed *Ae. comosa* (a M-genome species) within the B-genome lineage, not the expected D-genome lineage, contradicting those chloroplast-based phylogenetic inferences^21^.

Chloroplast (cp) with maternal inheritance can simplify the sequence-based evolutionary inferences of polyploid wheats, but informative inferences require a complete and accurate cp genome sequence because of the low nucleotide substitution rates in cp genomes compared to nuclear genomes^22–24^. Since 2002 when the first complete wheat cp genome sequence^25^ was released, efforts have been directed toward sequencing cp genomes of the complex^26–29^. With the advances in next-generation sequencing and the development of useful bioinformatics tools^30,31^, such genome sequencing has been more feasible than before^32,33^ and more cp genomes of polyploid wheats and their wild relatives are expected to be sequenced. So far, 95 complete, circular cp genome assemblies representing 10 *Aegilops* and 15 *Triticum* species and subspecies have been published in the National Center for Biotechnology Information (NCBI) database (Table S1). However, no specific efforts have been made to compare and characterize the published cp genomes of the complex^21,28^.

The objectives of this research were to (1) sequence, assemble and annotate the 16 cp genomes representing five *Aegilops* and 11 *Triticum* species and subspecies; (2) analyze and characterize the assembled cp genomes and those complete cp genomes published in NCBI database; and (3) infer and date the maternal divergence of the *Triticum–Aegilops* complex. It is my hope that this research would generate useful information for better understanding of the cp genome evolvability and maternal divergence of the *Triticum–Aegilops* complex.

## Materials and methods

### Plant material

This study consisted of 16 accessions of known species identity acquired from the Plant Gene Resources of Canada (PGRC) wheat collection on December 16, 2011. The acquisition was made for public good research following the Standard Material Transfer Agreement of the International Treaty on Plant Genetic Resources for Food and Agriculture. These accessions were selected to represent five *Aegilops* and 11 *Triticum* species and/or subspecies (Table 1, Table S2) and different nuclear genomes of variable ploidy (i.e., 2 ×, 4 × and 6 ×). The selected accessions were originally collected or donated from 10 countries and consisted of wild materials, breeding lines and cultivars. I also made an extra effort to identify the selected materials by growing the plants in a greenhouse and verifying the morphological characters by following taxonomic keys^4^. Also, the selected accessions were part of 7000 PGRC genebank accessions that were documented as herbarium specimens at the AAFC National Collection of Vascular Plants (DAO), Ottawa (Axel Diederichsen, personal communication).

**Table 1:** List of 16 samples representing five *Aegilops* and six *Triticum* species and their chloroplast genome assemblies, annotations and GenBank accessions.

Species	NG	PGRC Acc#	Raw Reads	CPG Size (bp)	CPG Region (bp)				All Genes	tRNA Genes	Pseudo Genes	NCBI Acc#
					LSC	IRb	SSC	IRa				
*Ae. comosa*	M	CN034253	48,98,139	1,36,247	80,497	21,491	12,770	21,489	130	38	3	**MG958548**
*Ae. longissima*	SI	CN108024	40,35,549	1,36,762	81,030	21,479	12,776	21,477	131	39	4	MG958549
*Ae. speltoides*	B	CN108018	23,97,826	1,35,982	80,214	21,489	12,792	21,487	130	38	3	MG958553
*Ae. tauschii*	D	CN034229	42,49,172	1,35,502	79,766	21,483	12,772	21,481	130	38	3	MG958544
*Ae. umbellulata*	U	CN108095	34,43,538	1,36,743	80,988	21,489	12,779	21,487	130	38	3	**MG958547**
*T. aestivum*	AuBD	CN011189	56,32,473	1,35,766	80,003	21,487	12,791	21,485	130	38	3	MG958554
*T. aestivum *ssp*. spelta*	AuBD	CN012261	39,02,845	1,35,819	80,054	21,488	12,791	21,486	130	38	3	MG958556
*T. monococcum *ssp*. aegilopoides*	Am	CN034176	44,75,344	1,36,820	81,048	21,484	12,806	21,482	130	38	3	**MG958551**
*T. monococcum *ssp*. monococcum*	Am	CN052948	36,92,717	1,36,758	80,986	21,484	12,806	21,482	130	38	3	MG958558
*T. timopheevii* ssp*. armeniacum*	GAu	CN034138	45,96,575	1,36,757	80,984	21,484	12,807	21,482	130	38	3	MG958559
*T. timopheevii *ssp*. timopheevii*	GAu	CN001847	27,34,120	1,36,028	80,257	21,495	12,789	21,487	131	39	4	MG958546
*T. turgidum *ssp*. dicoccoides*	AuB	CN107962	28,12,946	1,35,786	80,021	21,488	12,791	21,486	130	38	3	MG958552
*T. turgidum *ssp*. dicoccum*	AuB	CN011272	64,66,083	1,35,775	80,008	21,489	12,791	21,487	130	38	3	**MG958550**
*T. turgidum *ssp*. durum*	AuB	CN041178	48,55,401	1,35,772	80,005	21,489	12,791	21,487	130	38	3	MG958545
*T. urartu*	Au	CN038614	37,55,335	1,36,749	80,962	21,484	12,821	21,482	129	37	3	MG958555
*T. zhukovskyi*	GAmAu	CN036212	15,66,335	1,36,028	80,257	21,495	12,789	21,487	130	38	3	**MG958557**

The new circular chloroplast genome assemblies with annotations are highlighted in bold for NCBI accessions.

*NG* nuclear genome designation, *PGRC* Plant Gene Resources of Canada, *Acc*# accession number, *CPG* chloroplast genome, *LSC* large single copy, *SSC* small single copy, *IRa and IRb* two inverted repeat regions, *NCBI* National Center for Biotechnology Information.

About 300 seeds from each accession were planted on September 19, 2012 in a 15 cm pot and grown for 8–10 days in the greenhouse at the Saskatoon Research and Development Centre of Agriculture and Agri-Food Canada. The plants were incubated in the dark for 48–72 h to decrease the amount of starch stored in the leaves for reduced shearing of the chloroplast membranes in subsequent steps^34^. Tissue was collected from all 300 seedling leaves, totaling up to 15 g, and washed in cold water. Leaves were cut into 1 cm pieces with scissors, snap frozen with liquid nitrogen in a − 20 °C mortar, and then ground to a fine powder. Ground samples, while still frozen, were transferred to 50 mL conical-bottom centrifuge tubes, cooled on dry ice, and then stored at − 80 °C, for up to 1 week.

### DNA extraction and MiSeq sequencing

Plastid DNA isolation was performed in October 2012 following the method of Shi et al*.*^34^ and optimized using the cp DNA extraction protocol developed by Diekmann et al*.*^35^. All the procedures were carried out on ice or at 4 °C with buffers prechilled to 4 °C. The enriched cp pellet was allowed to thaw at room temperature, and DNA was extracted using the Qiagen DNeasy Plant Mini kit standard method on a QIAcube robot (Qiagen, Mississauga, Canada) and eluted in 1/3 × Qiagen AE buffer (3.33 mM Tris–Cl, 0.17 mM EDTA, pH9.0). DNA samples were quantified using the Quant-iT PicoGreen dsDNA Assay Kit (Life Technologies, Burlington, Canada). Final DNA yields ranged from 0.2 to 4.3 ng/µl and were diluted to 0.2 ng/µl with 10 mM Tris–HCl, with a pH of 8. The acquired cp DNAs were subjected to genomic DNA library preparation with a Nextera XT DNA Library Preparation Kit (Illumina, San Diego, USA) which uses a tagmentation step to produce DNA fragments of length ranging from 250 to 1000 bp and averaging roughly 500 bp. Four MiSeq runs, each with four libraries and 2 × 250 bp paired-end reads, were performed in January-March 2013 to generate 16 forward and 16 reverse FASTQ files. All raw reads were deposited into NCBI under the BioProject PRJNA433726 (Table 1, Table S2).

### Chloroplast genome assembling and annotation

Effort was initially made in 2013 to assemble the generated cp sequences following a reference-guided approach, as there was one published circular wheat cp genome sequence^25^. However, such effort did not generate satisfactory assemblies. New effort was made in 2017 to conduct de novo assemblies by generating contigs and scaffolds as MiSeq sequence read lengths of 250 bp can allow for the generation of better contigs and scaffolds^36^. All raw sequence reads were cleaned first with cutadapt^37^ to remove sequence adapters and to perform quality trimming. Partial Nextera adapter sequence ‘AGATGTGTATAAGAGACAG’ was used to trim the raw sequence reads. All the sequence reads with both quality lower than a Phred score of 15 and sequence shorter than 150 bp were discarded. Clean sequence reads were assembled using SPAdes v3.11.1^38^ for the circular cp genome assembly in the paired-end mode. Preliminary assembling was performed to evaluate the smallest number of contigs and the longest scaffold size by a series of combinations of different coverages and k-mer sizes. This evaluation concluded that the k-mer size should be set to 127, and the coverage set to 1000 fold. A cp genome has four genomic regions: large single copy (LSC), small single copy (SSC), and two inverted repeat regions (IRa and IRb), and the major gaps are normally located at the junctions of these four regions. To fill the gaps, the four junction sequences were utilized and obtained from the alignments of the scaffolds with their closely related species, including wheat (*T. aestivum*, NCBI Reference Sequence: AB042240.3), bent grass (*Agrostis stolonifera*; NCBI Reference Sequence: NC_008591.1), and ryegrass (*Lolium perenne*; NCBI Reference Sequence: NC_009950.1) cp genomes. Each of the four junction sequences (ranging between 540 and 700 bp) containing both IR and another (either LSC or SSC) structure fragment was used as a bait to screen for reads for further gap sequence recovery. The selected reads from BLAST were also used to link adjacent structure fragments. The additional gaps located within the scaffolds of some samples were similarly filled with the assistance of the bait sequences acquired from cp genomes of their closely related species with sequences at the same locations.

Gene annotations of the 16 cp genomes were made using the online DOGMA program^39^, along with the cp genome annotations of wheat (NCBI Reference Sequence: AB042240), ryegrass (NCBI Reference Sequence: NC_009950), and bent grass (NCBI Reference Sequence: NC_008591.1). Manual curation was also made for the variations within coding genes, such as rRNA and tRNA, based on multiple sequence alignments with their closely related species in the Triticeae tribe. The annotated cp genome sequences were deposited into and published in the NCBI database (Table 1) and formed as part of the published 95 circular cp genome sequences listed in Table S1.

### Acquisition of published cp genome sequences

Extra efforts were made to acquire those *Triticum*–*Aegilops* cp genomes published in the NCBI database for comparative analyses of sequence variation, selection pressure and maternal phylogeny. Such comparative analyses should yield extra information about the impacts of sample size on the specific analysis. As of March 23, 2021, there were 95 complete, circular *Triticum–Aegilops* cp genomes published in NCBI database, including those generated from this study, and these cp genomes represented 10 *Aegilops* and 15 *Triticum* species and subspecies (Table S1). An alignment analysis as described below revealed 22 of them had sequence heterogeneity and/or quality issues that might be associated with sequencing and/or genome assembling. Also the genome assembly for *T. aestivum* cultivar ‘Chinese Spring’ (CM022232.1) had no gene annotation. Consequently, these 23 cp genomes were excluded from further analyses. For ease of description and interpretation, the remaining 72 published cp genome sequences are named as 72cpgs, while the 16 cp genome sequences generated in this study are designated as 16cpgs. To enhance the inference of maternal divergence with respect to the role of *Ae. mutica* (or *Amblyopyrum muticum*)^19^, incomplete cp genomes of six *Ae. mutica* samples^21^ published in NCBI database were also acquired and combined with 72cpgs to make a specific dataset as 78cpgs for comparative phylogenetic analysis.

### Comparative genomic analysis

The comparative genomic analysis was made first on 16cpgs by generating and comparing their circular maps using GenomeVx^40^ and Circos version 0.69-4^41^. The maps were merged in Inkscape version 0.92 (https://inkscape.org). The second analysis of 16cpgs was conducted using mVISTA^42^ and merging the output figure with the GNU Image Manipulation Program version 2.8.20 (http://www.gimp.org) to identify the genomic regions with substantial variability among 16cpgs, using the *T. aestivum* cp genome assembly as an internal reference. Further alignment analysis of all 95 published cp genome sequences was also made using MAFFT^43^ with default options to compare the genomic structures and assess the quality of genome assemblies.

### SNP, SSR and diversity analysis

The SNP calling was performed using SNP-sites^44^ with the default options based on the multiple sequence alignments (MSA) of 16cpgs. MAFFT was used to generate MSA data with the FFT-NS-i × 1000 alignment algorithm. Consensus sequence was obtained using CONS of the EMBOSS software suite^45^ and annotated with GeSeq^46^, and SNP annotation was conducted using SnpEff^47^. The SnpEff analysis assumed a linear genome sequence from the beginning of the LSC region to the end of the IRa region. SSRs present among 16cpgs were analyzed using MISA^48^, with the following default setting of minimum number of repeats to 10, 6, 5, 5, 5, and 5 for mono-, di-, tri-, tetra, penta-, and hexa-nucleotides, respectively. To estimate nucleotide diversity across 16 samples, the sliding window diversity analysis was made using DnaSP v6^49^ with a sliding window of 2000 bp and step size of 200 bp. These three types of analysis were also performed on 72cpgs.

### Selective pressure analysis

Several site models (M0, M1, M2, M3, M7, and M8), implemented in codeml of PAML v 4.9i^50^, were applied to estimate the Ka/Ks and ω values, considering F3X4 codon frequencies, from 16cpgs. Coding protein sequences for all cp genes were extracted from each assembled cp genome. MAFFT, with the default options, was used to align the protein sequences of all annotated cp genes and aligned protein sequences were translated to nucleotide sequence using BACKTRANSEQ of the EMBOSS software suite^45^. The phylogenetic tree of 16 samples, without tree-branch lengths, was obtained from the phylogenetic analysis described below. Four nested site models (M3 vs. M0; M2 vs. M1; M8 vs. M7; and M8a vs. M8) were evaluated by log-likelihood ratio tests (LRT). The positively selected sites were analyzed by Naïve Empirical Bayes (NEB) analysis and Bayesian Empirical Bayes (BEB) analysis. Extra effort was also made to perform a mixed-effects maximum likelihood analysis of natural selection on individual codons of the annotated cp genes using HyPhy MEME method^51^ with the default options. The same PAML codeml and HyPhy MEME analyses were conducted on 72cpgs.

### Phylogenetic analysis

The phylogenetic analysis of 16cpgs and 78cpgs started with the search for the best fit nucleotide substitution model using SMS software^52^. The general time reversible (GTR) model was found to be the best fit based on the Akaike information criterion. Based on the GTR model, further analyses were performed using BEAST v2.0.342^53^ with the extra considerations of K80 (HKY) and two clock models. Comparing the resulting Bayesian maximum clade credibility (MCC) trees with respect to tree topology and branch support revealed the GTR model generated essentially the same tree topology but with higher branch support than the HKY model. The final BEAST settings were obtained as: the substitution model was GTR; clock model was relaxed clock exponential; tree prior was Yule model; the outgroup was set for wild barley for monophyletic analysis with prior of Inverse gamma; and the rest of the options were kept with default values with a MCMC chain length of 100 million. The convergence of parameters among runs was evaluated visually using Tracer v.1.649^54^. The output tree files were loaded into TreeAnnotator in the BEAST package with the default options: 10% burnin and 0.50 posteriori probability limit and median node heights to combine and construct a MCC tree. The Figtree_v1.4 software (http://tree.bio.ed.ac.uk/software/figtree/) was used to display the MCC tree with the posterior probability as branch support and with node labels with time scale to root age of 11.6 Mya as estimated using nuclear genes for the wheat-barley divergence^14^.

To verify the maternal phylogeny of 16cpgs, further BEAST-based analysis was made using *Secale cereale* (NCBI KC912991) as outgroup and the obtained MCC tree was calibrated at the root age of 6.7 Mya as estimated by Chalupska et al*.*^14^ using nuclear genes. To assess the variability of lineage ages in both barley- and rye-rooted MCC trees, the age ranges of wheat-barley and wheat-rye lineages (9.7–12.2 and 3.4–4.1 Mya, respectively) inferred by Bernhardt et al*.*^21^ from Triticeae tribe-wide cp genes were applied to calibrate various lineages reflecting maternal divergences of 2 ×, 4 × and 6 × species in the *Triticum*–*Aegilops* complex.

The same phylogenetic analysis using BEAST with the same settings was repeated on 78cpgs using both wild barley and rye as outgroup, respectively. The same effort was also made to date the major lineages of the two 78cpgs based MCC trees based on the age estimates both from nuclear and cp genes.

### Research involving plants

The use of plants in the present study complies with international, national and/or institutional guidelines.

### Ethical standards

Experimental research on the plants and the writing process of this manuscript comply with the current laws of Canada.

## Results

### Sequencing and genome assembly

Four MiSeq runs generated a total of 69 million sequence reads for the 16 *Triticum–Aegilops* samples, each having an average of 4.3 million sequence reads (Table S2). After removing sequence reads of poor quality (Q < 15 and read length < 150 bp), an average of 92.2% high-quality sequence reads were obtained for these samples. Thus, each sample still had a sequence length ranging from 391 to 1617 Mbp, with an average of 977 Mbp, corresponding to an approximately 6417 × to 24,004 × cp genome coverage. Such high genome coverages made the cp genome assembly simpler, with the smallest numbers of contigs and scaffolds (Table S2) under proper k-mer coverage and size setting.

De novo assembly with the paired-end sequence reads from each sample generated three major scaffolds, as expected with two inverted repeats (IRs), with the exception of five scaffolds for the sample of *Ae. comosa*. All the circular *Triticum–Aegilops* cp genomes consisted of four typical DNA fragment structures: LSC, SSC, IRa and IRb. The cp genome sizes ranged from 135,502 (*Ae. tauschii*) to 136,820 bp (*T. monococcum* ssp*. aegilopoides*), with an average of 136,206 bp (Table 1). The average sizes in base pair for LSC, SSC, and IR among the 16 cp genomes were 80,443, 12,791, and 21,487, respectively (Table 1). The average of GC contents for these cp genomes ranged from 38.25 to 38.30%, with an average of 38.27% (Table S2). The circular cp genomes generated for *Ae. comosa*, *Ae. umbellulata*, *T. monococcum* ssp. *aegilopoides, T. turgidum* ssp*. dicoccum* and *T. zhukovskyi*, as highlighted in Table 1, were new additions to the sequenced cp genomes with gene annotations for the *Triticum–Aegilops* complex^21,27,28^.

### Chloroplast genome gene annotation

The gene annotations revealed 129–131 genes, including 84 coding genes, 37–39 tRNAs, eight rRNAs, and three or four pseudogenes for these assayed cp genomes (Table 1, Table S3). However, some variation was observed with the *T. urartu* cp genome having 129 genes with only 37 tRNAs and with the cp genomes of *Ae. longissimi* and *T. timopheevii* ssp*. timopheevii* having 131 genes with 39 tRNAs and four pseudogenes. Specifically, three pseudogenes (*rps*12, *trn*T, *trnf*M) were present in all the 16 cp genomes, while the *trn*T pseudogene only occurred twice in those of *Ae. longissima* and *T. timopheevii* ssp*. timopheevii.* Note that *rpl*23, *trn*T and *trnf*M still have functional copies in addition to the pseudogene and that *T. urartu* cp genome had only one copy of *trn*G with no introns.

### Comparison of genomic structures

**Figure 1:**
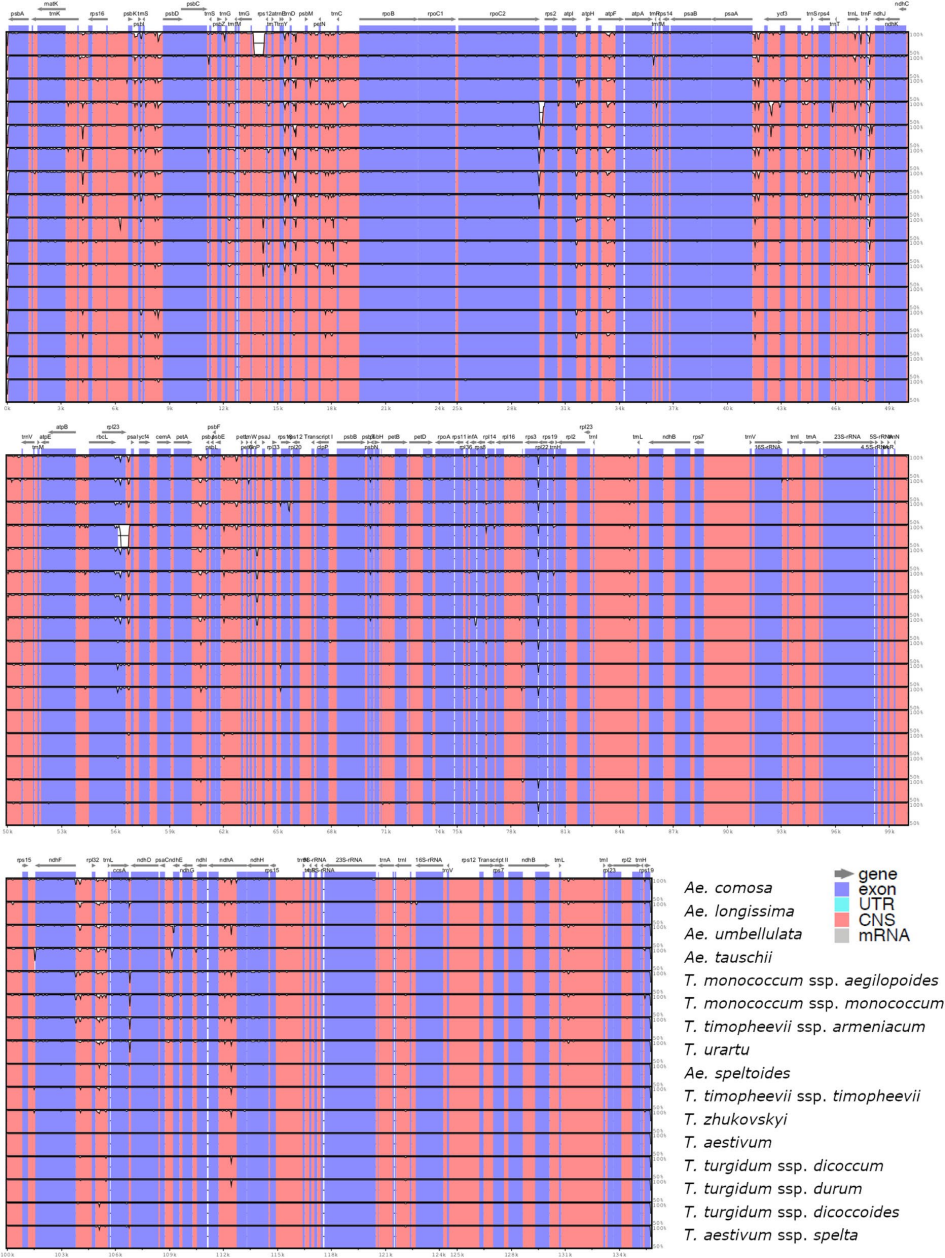
Percent identity plot of 16 complete *Triticum* and *Aegilops* chloroplast genome assemblies, using *T. aestivum* as internal reference. The vertical order of samples (or rows) followed the tree topology shown in Fig. 3. Vertical scale indicates the percentage of identity ranging from 50 to 100%. Coding regions are in blue and non-coding regions are in orange. Note that the plot was generated using mVISTA^42^ (http://genome.lbl.gov/vista/mvista/about.shtml) and merging the output figure with the GNU Image Manipulation Program version 2.8.20 (http://www.gimp.org).

Analyzing the mVISTA percent identity plot of 16cpgs revealed several conservative features of genomic variation, as illustrated in Fig. 1. First, no marked differences in genomic structure and gene arrangement were observed. The revealed gene arrangement was consistent with those gene maps illustrated in Fig. S1 of the five cp genomes representing the nuclear genomes of 2 ×, 4 × and 6 × species. Second, the degree of similarity between any two of the 16 cp genome sequences was generally high (or larger than 90%), particularly for those *Triticum* cp genomes. However, there were two exceptions. *Ae. comosa* cp genome had one large gap of 543 bp in the conserved non-coding sequence (CNS) region before *rps*12 gene. *Ae. tauschii* cp genome had two large gaps: one of 174 bp in the CNS region before *rps*2 gene and another of 1059 bp in the 3' end of *rpl*23 gene and a part of the CNS region. These two gaps helped to explain why *Ae. tauschii* cp had the smallest genome size of 135,502 bp (Table 1). Further MSA analysis of three incomplete *Ae. comosa* and 18 complete *Ae. tauschii* cp genomes published in the NCBI database confirmed the presence of these three gaps in these two species. Third, most of the nucleotide variations across 16cpgs were located in intergenic regions, mainly reflecting those variations among the *Aegilops* cp genomes. Fourth, there were no specific variations in genomic structure and gene arrangement unique to each ploidy level.

The MSA analysis of 95 published circular cp genome sequences (Table S1) revealed 22 of them had many gaps of different sizes (up to 600 bps), particularly in the IRb and IRa regions, and many extra fragments of up to 300 bps at the beginning and the end of the genome sequences, while 73 of them had the exact start and ending of the whole genome with gaps largely of size less than 30 bps within the genome. To verify the detected sequence heterogeneity, a phylogenetic analysis of the 95 cp genome sequences as described below was made and many of those 22 cp samples were found to reside with the samples of different species and/or subspecies. Further visual assessment of the 72cpgs MSA file also showed some released genome sequences of the same *Triticum* species or subspecies varied considerably in substitution. These findings together indicated the presence of sequence heterogeneity or quality issues among the published cp genome sequences.

### Sequence variation and divergence

The SNP calls based on the alignments of 16cpgs and 72cpgs revealed a total of 871 and 1183 SNPs for these *Triticum–Aegilops* species, respectively, and these detected SNPs also had a total count of possible effects as 16,313 and 21,775, respectively, as predicted with SnpEff (Table 2). The SNPs were widely distributed over the cp genomes, but a unique feature was observed that more than 80% of the detected SNPs resided on the downstream (41.18–41.70%) and upstream (39.52–40.01%) gene regions. Only 7.97–8.85% SNPs were located on the intragenic regions and 2.37–2.48% SNPs on the intergenic regions, while 4.25–4.72% specifically on the introns. These SNPs were also predicted to be associated with missense (2.16–2.37%), nonsense (0.40–0.41%), and silent (0.53–0.65%) mutations. Note that the gene regions defined in SnpEff annotation differ from those four genomic regions of a cp genome (LSC, SSC, IRb and IRa), as explained in Table 2. Moreover, similar results of SNP annotations were found between 16cpgs and 72cpgs.

**Table 2:** SNP detection and annotation based on the consensus sequences of the 16 and 72 chloroplast genomes of the *Triticum–Aegilops* complex using SnpEff^47^.

Type	Count	Percent	Count	Percent
	*16 cp in this study*		*72 cp published*	
Total SNPs	871		1183	
Total possible effects	16,313		21,775	
Downstream gene variant	6803	41.70	8967	41.18
Upstream gene variant	6447	39.52	8712	40.01
Intragenic variant	1443	8.85	1736	7.97
Intron variant	693	4.25	1027	4.72
Intergenic region	404	2.48	516	2.37
Missense variant	353	2.16	516	2.37
Synonymous variant	86	0.53	142	0.65
Non coding transcript exon variant	17	0.10	61	0.28
Stop lost	23	0.14	40	0.18
Stop gained	29	0.18	35	0.16
Stop retained variant	13	0.08	15	0.07
Non coding transcript variant	2	0.01	4	0.02
5 prime UTR variant			2	0.01
Splice region variant			2	0.01

Note that the gene regions defined in SnpEff annotation differ from those four regions of cp genome structure (LSC, SSC, IRb and IRa). Up/Downstream gene means the distance to the first/last codon of the gene; Intergenic region mean the distance to the closest gene; and Splice region means the region of the splice site, either within 1–3 bases of the exon or 3–8 bases of the intron.

The SSR analysis revealed a total of 459 and 1881 SSRs for 16cpgs and 72cpgs, respectively (Table S4). The SSR counts per sample ranged from 20 (two samples of *Ae. tauschii*: KU198486 and KU198482) to 35 (*T. monococcum* ssp. *monococcum*: MG958558), with an average of 26.1. However, more SSRs per sample seemed to harbor in 16cpgs than the other 56 cp genomes, when compared with the averages of 28.7 and 25.4, respectively. The SSR motifs consisted of 13 poly-A/T with 10–26 repeats; two poly-C/G with 10–11 repeats; one poly-AT/TA with six repeats; and one poly-AAT/ATA with five repeats (Table S4). Three abundant SSR motifs were poly-A/T with 10, 11, and 12 repeats. However, no SSR motifs for tetra, penta-, and hexa-nucleotides were identified.

**Figure 2:**
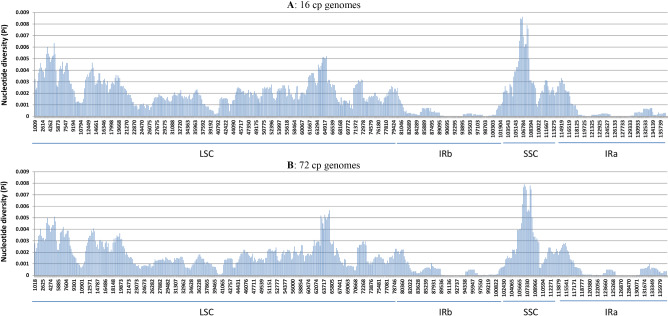
Nucleotide diversity (Pi) from the sliding window analysis of the 16 complete *Triticum* and *Aegilops* chloroplast genome assemblies generated in this study (**A**) and published 72 circular chloroplast genome sequences (**B**) (window length: 2000 bp, step size 200 bp). X-axis: position of the window midpoint, Y-axis: nucleotide diversity within each window. Note that the plots were generated in Microsoft Excel based on the outputs from DnaSP 6 software^49^.

The sliding window analysis of nucleotide diversity in 16cpgs showed that the genomic region of SSC had the highest nucleotide diversity, followed by LSC, while the two repeat regions (IRa and IRb) had the lowest nucleotide diversity (Fig. 2A). Three specific genome positions with the highest diversity were the sliding windows near 106,578, 5067, and 65,321 (Fig. 2A), corresponding to the sequence region for genes *ccs*A, *rps*16, and *rpl*33, respectively. Further assessment of nucleotide diversity variation in 72cpgs revealed the same patterns of nucleotide diversity variation across the cp genomes as observed in 16cpgs, although the extent of diversity varied slightly for some sliding windows (Fig. 2B).

### Selective pressure analysis

The analysis of positive selection at individual codons of 84 coding genes was performed on 16cpgs and 72cpgs using two methods (PAML codeml and Hyphy MEME). The codeml analyses generated the likelihood ratio tests for three models (M2a vs. M1a; M8 vs. M7; and M8 vs. M8a) for each set of samples and revealed that 30 and 141 codons were under positive selections for 16cpgs and 72cpgs, respectively, based on Bayesian Empirical Bayes tests (Table S5). The MEME analyses identified 3 and 19 sites displaying episodic diversifying selection for 16cpgs and 72cpgs, respectively. Specifically, these significant codons were associated with 24 of the 84 coding genes located in LSC, SSC and IRa regions (Table 3). Increasing sample size from 16 to 72 also increased the number of the significant codons detected from 30 to 141 and associated genes identified from 15 to 23. The proportions of codons showing positive selection for these genes were small, ranging from 0.001 to 0.178 out of expected total codons for a gene and averaging 0.024. Three genes showing the highest proportions of significant codons were *ndh*H (0.178) in the SSC region, *rps*15 (0.133) and *rpl*23 (0.086) in the IRa region. Also the genes *rpo*C2 and *mat*K in the LSC region displayed more significant codons, although the proportions were only 0.010.

**Table 3:** The extents of codons showing positive selection in the chloroplast (cp) genes in two sets of samples (16 cp genome assemblies generated in this study and published 72 cp genome sequences) obtained from the tests by two methods (PAML codeml and Hyphy MEME).

Sample	Total codons	CPG region	72 cp			16 cp	
Method			Codeml		MEME	Codeml	MEME
Gene			Codon count	Proportion	Codon count	Codon count	Codon count
*psb*A	353	LSC	1	0.003	1		
*mat*K	512	LSC	5	0.010	3	5	
*rpo*B	1076	LSC	1	0.001	1	1	
*rpo*C1	683	LSC	4	0.006		2	
*rpo*C2	1479	LSC	15	0.010	3	7	1
*atp*I	247	LSC	1	0.004			
*atp*A	504	LSC	1	0.002		1	
*paf*I	170	LSC	1	0.006	2		
*rps*4	201	LSC			1		
*ndh*K	245	LSC	2	0.008	1	2	1
*atp*E	137	LSC	1	0.007	1	1	
*atp*B	498	LSC	1	0.002		1	
*rbc*L	477	LSC	1	0.002			
*pet*A	320	LSC	1	0.003		1	
*psb*H	73	LSC	1	0.014		1	
*rpl*14	123	LSC	1	0.008		1	
*ndh*F	739	SSC	2	0.003	1	2	
*ccs*A	322	SSC	2	0.006	1	2	1
*ndh*D	500	SSC	1	0.002	1		
*ndh*A	362	SSC	3	0.008	3	1	
*ndh*H	393	SSC	70	0.178		2	
*rps*15	90	IRa	12	0.133			
*rps*7	156	IRa	6	0.038			
*rpl*23	93	IRa	8	0.086			
Sum/Average	9753		141	0.024	19	30	3
Total genes	24		23		12	15	3

Note that the total codon count for each associated gene was obtained from the coding protein sequences of *T. aestivum* (MG958554).

*CPG* chloroplast genome.

### Maternal phylogenetic trees

**Figure 3:**
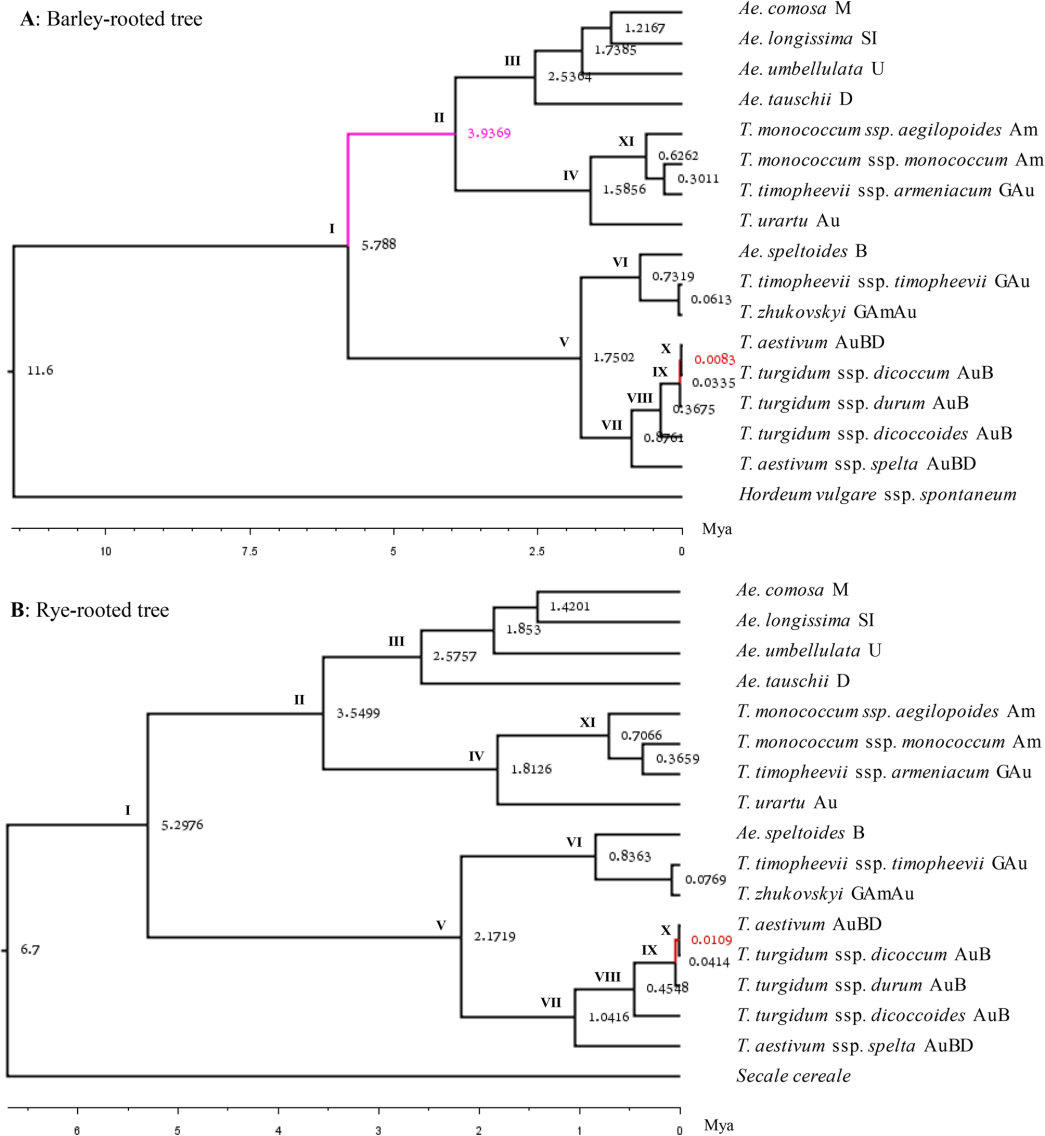
Maternal phylogenetic trees of 16 samples representing six *Triticum* and five *Aegilops* species with node age and support, as inferred from chloroplast sequence variations using BEAST software. The top (**A**) and bottom (**B**) trees were rooted with wild barley (*Hordeum vulgare* ssp. *spontaneum*) and rye (*Secale cereale*), respectively. The node ages were calibrated with the root ages in Mya following the related age estimates by Chalupska et al*.*^14^. All the nodes had supports with posterior probability of 0.99 or higher, except one node in purple and two nodes in red with the posterior probabilities of 0.89 and 0.33, respectively. Eleven major nodes are labeled and the sample is labeled with its nuclear genome designation.

Two rooted Bayesian MCC trees of 16cpgs with nodal supports and ages were illustrated in Fig. 3. The trees shared the same topology. All the nodes had supports with posterior probability of 0.99 or higher, except one node in purple and two nodes in red, with the posterior probabilities of 0.84 and 0.33, respectively. Eleven major nodes of interest are labeled to represent the major divergences among the *Triticum–Aegilops* complex with 2 ×, 4 × and 6 × species. Analyzing the MCC trees revealed some patterns of maternal evolution in the *Triticum–Aegilops* complex. First, maternal divergence of the *Triticum–Aegilops* complex had three deep lineages (III, IV and V) representing three important 2 × species with the nuclear genomes A (*T. urartu*), B (*Ae. speltoides*) and D (*Ae. tauschii*). *Ae. tauschii* represented the lineage III with four *Aegilops* species, and did not show it as an immediate maternal donor to any assayed 4 × or 6 × species. *T. urartu* represented the lineage IV and showed its close relation to einkorn wheat (*T. monococcum*) (or the lineage XI) and wild hulled wheat (*T. timopheevii* ssp. *armeniacum*). *Ae. speltoides* was located within the lineage V with 4 × and 6 × wheat species, but it was closely related to domesticated hulled wheat (*T. timopheevii* ssp. *timopheevii*) and Zhukovsky's wheat (i.e., lineage VI). Second, *T. aestivum* ssp. *spelta* was closely related to emmer, durum and bread wheats (as shown in the lineage VII). It seemed that wild emmer (or lineage VIII) contributed as the maternal donors to emmer, durum and bread wheats and that emmer and bread wheats (or lineage X) shared the maternal progenitor of a cultivated durum wheat (or lineage IX).

**Figure 4:**
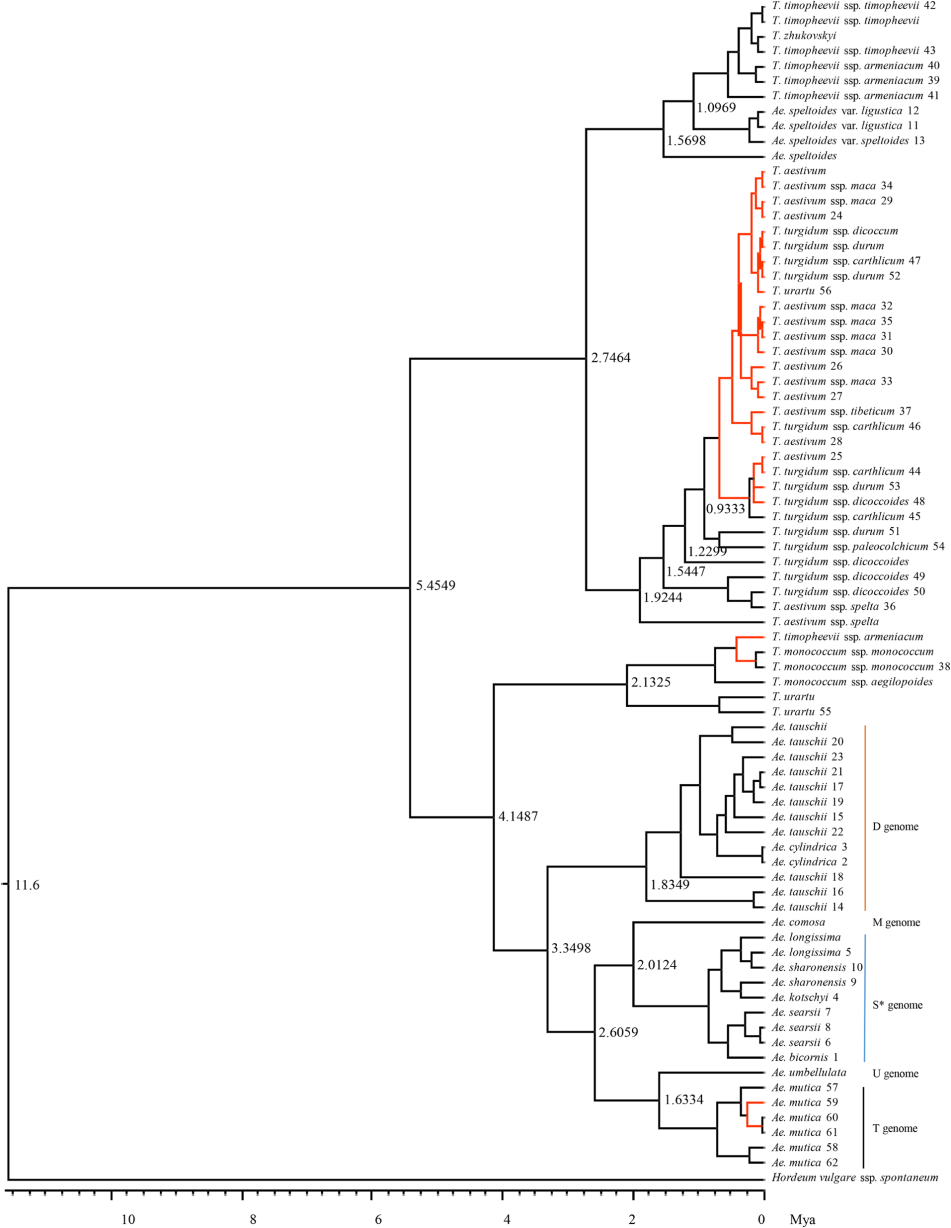
Bayesian maximum clade credibility trees of published 72 complete and six incomplete chloroplast genomes representing six *Triticum* and 11 *Aegilops* species with nodal support (in posterior probability) and outgroup of *Hordeum vulgare* ssp. *spontaneum*, as inferred from chloroplast sequence variations using BEAST software. A sample with a number after its species name was published by others and a sample without the number label was assembled from this study (see Table S1). The nodes with a posterior probability of 0.90 or less are highlighted in red. The node ages were calibrated with the root ages in Mya following the related age estimates by Chalupska et al*.*^14^. Some lineage ages are shown, along the divergence time axis at the bottom of the figure.

Further analysis of the barley-rooted MCC trees of 78cpgs representing 11 *Aegilops* and 15 *Triticum* species and subspecies revealed essentially the same tree topology (Fig. 4) as those inferred from 16cpgs (Fig. 3). For example, the same three deep lineages each representing a diploid species with nuclear A, B, or D genome were found in the rooted MCC tree (Fig. 4). The D-genome lineage^18^ was comprised of the D + S* + M + U + T genome species, similar to those described by Li et al*.*^11,55^ where *Ae. searsii* and *Ae. sharonensis* retained its S* group, *Ae. comosa* represented the M group, *Ae. umbellulata* the U group, and *Ae. mutica* the T group. *T. urartu* showed the same close relationships to einkorn wheat (*T. monococcum*) and wild hulled wheat (*T. timopheevii* ssp. *armeniacum*). However, the 78cpgs based MCC tree carried more nodes with less nodal support toward hexaploid wheat (Fig. 4) than those observed in Fig. 3 for 16cpgs. The rye-rooted MCC tree (Fig. S2) showed essentially the same topology as the barley-rooted MCC tree of 78cpgs (Fig. 4), but revealed slightly more nodes with less nodal support, particularly toward hexaploid wheat.

### Dating major lineages

Divergence dating was made on the barley-rooted and rye-rooted MCC trees of 16cpgs. The node ages of both trees were shown in Fig. 3 and they were calibrated with the root ages of wheat divergence from barley at 11.2 Mya and of wheat divergence from rye at 6.7 Mya, which were estimated from nuclear gene variation by Chalupska et al.^14^. Essentially both trees showed compatible ages of the corresponding nodes. For example, the divergence for the lineage I between the nuclear genomes A and B occurred at 5.3 Mya; for the lineage II between the nuclear genomes A and D at 3.6–3.7 Mya; for the divergence between wild and domesticated emmer (or lineage VIII) at 0.3282–0.4197 Mya; and for the lineage X between the emmer and bread wheats at 8200–11,200 years ago. An effort was also made to date major lineages calibrated with the cp gene based estimates of divergence age reported by Bernhardt et al*.*^21^. For the barley-rooted tree, cp gene based calibrations for node ages were compatible with those made using nuclear genes (Table 4). For example, the divergence for the lineage I between the nuclear genomes A and B occurred at 4.4–5.6 Mya and for the lineage X between the emmer and bread wheats at 6900–8700 years ago. For the rye-rooted tree, the calibration was made at the lineage of the genomes A and B, the divergence for the lineage V between AuBD and B occurred at 1.29–1.55 Mya, and the divergence for the lineage X between the emmer and bread wheats appeared at 7100–8700 years ago (Table 4). Thus, node age calibrations based on cp genes were compatible to those using nuclear genes. Based on the cp gene based calibrations, the divergence between domesticated and wild einkorn wheats (or lineage XI) occurred at 0.4525–0.6590 Mya, between domesticated and wild emmer wheats (or lineage VIII) at 0.2685–0.3451 Mya, and between durum and bread wheats (or lineage IX) at 0.0259–0.0326 Mya (Table 4).

**Table 4:** Estimated node ages with standard deviation (in parenthesis) for seven major lineages (I: AD-B, II: A-D, V: AuBD-B, XI: Einkorn, VIII: Emmer, IX: Durum-Bread, X: Emmer-Bread wheat) for barley- and rye-rooted trees.

Tree	Rooted age	Lineage						
		I: AD-B	II: A-D	V: AuBD-B	XI: Einkorn	VIII: Emmer	IX: Durum-Bread	X: Emmer-Bread
**Calibrated by nuclear genes**								
BrT	Mean 11.6	5.7880(3.3860)	3.9369(2.2385)	1.7502(1.2728)	0.6262(0.4363)	0.3675(0.2468)	0.0335(0.0401)	0.0083(NA)
RrT	Mean 6.7	5.2976(1.9577)	3.5499(1.4188)	2.1719(0.9709)	0.7066(0.3698)	0.4548(0.2743)	0.0414(0.0456)	0.0109(NA)
**Calibrated by cp genes**								
BrT	LB 9.7	4.8400(2.8314)	3.2921(1.8718)	1.4635(1.0644)	0.5236(0.3468)	0.3074(0.2064)	0.0281(0.0335)	0.0070(NA)
BrT	UB 12.2	6.0874(3.5612)	4.1405(2.3543)	1.8407(1.3387)	0.6586(0.4588)	0.3866(0.2595)	0.0354(0.0422)	0.0088(NA)
RrT	LB 4.3001	3.4000(1.2516)	2.2783(0.9071)	1.3939(0.6207)	0.4535(0.2364)	0.2919(0.1753)	0.0266(0.0298)	0.0070(NA)
RrT	UB 5.1854	4.1000(1.5093)	2.7474(1.0938)	1.6809(0.7484)	0.5469(0.2851)	0.3520(0.2114)	0.0321(0.0359)	0.0085(NA)

The lineages were defined in the maternal phylogenetic trees in Fig. 3. The calibrations were made at the root age in Mya estimated from nuclear genes by Chalupska et al*.*^14^ and chloroplast (cp) genes by Bernhardt et al*.*^21^.

BrT is barley-rooted tree. RrT is rye-rooted tree. LB is lower bound. UB is upper bound. The standard deviation was obtained based on the estimate of height_95%_HD and scaled with the root age, but it was not obtained for the lineage X (Emmer-Bread) due to little sequence variation and labeled as not available (NA).

Effort was also made to date the divergence of the barley-rooted and rye-rooted MCC trees of 78cpgs (Fig. 4, Fig. S2). Based on the same calibration with nuclear genes, both MCC trees displayed the older nodes of the same lineage than those for 16cpgs, particularly for those young lineages. For example, the age of the lineage V (for B-genome and hexploid wheat) for the barley-rooted MCC tree of 78cpgs was estimated to be 2.7464 Mya (Fig. 4), compared to 1.7502 Mya for the tree of 16cpgs. Such increase in node age was more obvious for the rye-rooted MCC tree (Fig. S2) where the lineage V was estimated to emerge 3.4782 Mya. The same pattern of increased node ages was observed for cp gene based calibrations (not shown). These results indicate the divergence dating was conditional on the sample size used.

## Discussion

The de novo assembling generated five new circular cp genome sequences with gene annotations (Table 1). The SNP analysis revealed that more than 80% of the detected cp SNPs were distributed on the downstream and upstream gene regions and only 2.78% or less SNPs were predicted to be deleterious (Table 2). The selection analysis yielded that a mild positive selection had acted on the annotated 84 coding cp genes (Table 3). The inferred phylogenetic trees showed the maternal divergence of the *Triticum–Aegilops* complex with three deep lineages each representing a diploid species with nuclear A, B or D genome (Figs. 3, 4). The divergence dating revealed that the maternal divergence between the emmer and bread wheats occurred at 8200–11,200 years ago (Table 4). These findings are significant for understanding the chloroplast genome evolvability and maternal divergence of the complex.

Characterizing the *Triticum–Aegilops* cp genomes revealed some interesting genomic characteristics as summarized here. First, no marked differences in cp genomic structure and gene arrangement were identified across 16cpgs, except for *Ae. comosa* cp genome with one indel and *Ae. tauschii* cp genome with two indels (Fig. 1). Second, the basic gene count in 16cpgs was 130, but *T. urartu* and *Ae. longissima* had 129 and 131 genes due to the change in one extra tRNA, respectively (Table 1). Third, more than 80% of the detected SNPs resided on the downstream and upstream gene regions and less than 3% SNPs were predicted to be detrimental (Table 2). Fourth, the patterns of SSR motif and repeat obtained were similar across 72cpgs, although the extents of SSRs per sample varied from 20 to 35 (Table S4). Fifth, the same genome-wide patterns of nucleotide diversity were observed either in 16cpgs or 72cpgs (Fig. 2). Sixth, the positive selection detected from both 16cpgs and 72cpgs was relatively weak acting on the annotated 84 coding genes (Table 3). These comparative genomic features enhanced our understanding of the *Triticum–Aegilops* cp genomes, particularly with respect to variability and evolvability.

More SNPs were detected in 72cpgs than 16cpgs, but the results of SNP annotation for the two sets of cp genomes were similar (Table 2). For example, 41.7% SNPs detected in 16cpgs were distributed on the downstream gene regions, while 40.18% in 72cpgs. It should be noted, however, that such SNP annotations provided only a general pattern of SNP distribution and predicted effects in the plastids of the *Triticum–Aegilops* complex, not specific to any of the analyzed cp genomes, as they were based on the consensus genomes and their gene annotations. Also, the consensus sequence and its gene annotation can vary in accuracy, largely depending on the number and quality of the analyzed cp genomes. Thus, caution is suggested for interpreting the predicted effects of specific SNPs. The SSR analysis of 72cpgs revealed the same patterns of SSR motif and repeats as reported by Tomar et al*.*^56^ for *T. aestivum* cp genomes only. However, our SSR results were more extensive in cp genomes and had more SSRs detected. For example, we detected 27 SSRs rather than those 25 SSRs reported in *T. aestivum* cp genomes and identified three SSRs unique to the durum wheat sample of KM352501.1 and one SSR unique to the bread wheat sample of MH051715.1 (Table S4). Thus, our SSR findings (Table S4) can serve as a useful genomic resource for the development of cp SSR markers to characterize alloplasmic lines and to identify wheat hybrids and wild relatives^56^.

To my knowledge, there has not been a report so far on specific genome-wide selection analysis of significant codons in the cp coding genes of the *Triticum–Aegilops* complex. The analysis of the selection pressure acting on 15,589 codons of the 84 coding genes in 72cpgs revealed 141 significant codons associated with 24 genes in three genomic regions (LSC, SSC and IRa) (Table 3). However, considering the proportion of the significant codons (0.9%) and the extent of the associated coding genes (28.6%), it seemed that the overall selection pressure acting on these chloroplast coding genes was relatively weak. This finding is consistent with the stable patterns of genome-wide nucleotide diversity (Fig. 2) observed with an increased sample size from 16 to 72 cp genomes and also with the extent (or less than 3%) of the detrimental SNPs predicted (Table 2). Together, these results indicate that the chloroplast genomes of the *Triticum–Aegilops* complex were largely stable genetically. Such genetic stability is somehow expected, given their short divergence with only approximate five million years^14,18,21^. However, it is worth noting that our selection analysis seemed to be associated with the sample size and method used (Table 3). The codeml analysis detected more selective codons than the Hyphy MEME method and more significant codons were found in 72cpgs than 16cpgs in both methods. Thus, further research is needed to understand these selection analyses for a better detection of significant codons. Also, it remains unknown why a few coding genes such as *ndh*H, *rps*15*, rpl*23, *rpo*C2 and *mat*K carried many significant codons, but it is possible that such significance was associated with the observations of abundant indels in the gene or nearby regions. For example, *ndh*H carried a 114-bp deletion in the *T. timopheevi* species^57^.

The rooted phylogenetic trees of 16cpgs shared essentially the same topologies as those for 78cpgs with respect to nuclear A, B and D genome species (Figs. 3, 4, Fig. S2). These cp-based MCC trees were consistent with our current knowledge of the maternal origin of polyploid wheats^58^. Our phylogenetic inference further supports that *Ae. speltoides* was the maternal donor of all polyploid wheats^26,59,60^. *Ae. tauschii* was not the maternal donor to any of the diploid, tetraploid and hexaploid wheats (see the lineage III of Fig. 3), which was compatible with the early nuclear-marker and cytogenetic findings reported by Dvorak et al*.*^61^ and Kerby and Kuspira^62^, respectively. The composition of the lineage III is consistent with those D + S* + M + U + T genome species (Fig. 4). Such lineage composition deviated more from those reported with nuclear sequences^18^, but was more compatible to those described by Li et al*.*^11,55^, thus supporting the hypothesis of nested rounds of hybridization events at the origin of the wheat D genomes. *Ae. mutica* remained in the D-genome lineage, which was consistent with the previous cp-based phylogenetic analysis^21^, but it was not phylogenetically close to the B-genome lineage as reported with nuclear sequence variations^19,63^, suggesting this species didn’t contribute its chloroplast to other polyploidy wheat species. *T. turgidum* ssp. *dicoccoides* might be the maternal progenitor of modern durum, emmer and bread wheats (as shown within the lineage VII of Fig. 3), and *T. turgidum* ssp. *dicoccum* was most likely the close maternal donor of bread wheat, which is consistent with those found from nuclear sequence signals^14^. *T. urartu* was the maternal donor of diploid einkorn wheat and tetraploid wild hulled wheat (*Triticum timopheevii* ssp. *armeniacum* (see the lineage IV of Fig. 3). These findings, together, are complementary to our current knowledge about maternal contributions of A, B and D donors to the origin of polyploid wheats.

It is generally difficult to date maternal divergence of the recently evolving species like the *Triticum–Aegilops* complex, mainly because the recently evolving species normally carry little cp substitution variation and little fossil evidence exists to support the age calibration. Our divergence dating, however, was based on the accumulated knowledge on the major lineages of the Triticeae tribe inferred previously from the evolutionary signals of both nuclear and cp genes^14,18,21^. The inferred maternal divergence of 16cpgs matched well with those previously reported^12,14,18,21^. For example, the maternal divergence between the nuclear genomes A and B occurred at 5.3 Mya and between the nuclear genomes A and D at 3.6–3.7 Mya (Fig. 3; Table 4). The finding that the divergence between wild emmer and bread wheats occurred at 0.32–0.42 Mya is compatible with those inferred from transposable elements and mutations^12^, and the maternal divergence between the emmer and bread wheats occurred at 8200–11,200 years ago (Table 4) is also consistent with those reported for wheat domestication in the Near East^64^. However, it is worth noting that the divergence dating may have overestimated the lineage ages as Middleton et al*.*^27^ indicated if multiple haplotypes and/or independent chloroplast lineages existed in the species complex. The presence of low nucleotide substitutions could have also underestimated the ages of young lineages^21^. Thus, it is important to consider the age estimations from multiple evolutionary signals. The age calibrations from both nuclear and chloroplast genes yielded compatible age estimates on these lineages, although the latter showed slightly lower estimates than the former (Table 4), and thus are useful for further age calibrations of these recently evolving lineages in the *Triticum–Aegilops* complex. However, we also found the lineage ages increased with the increased sample size, as shown with 16cpgs (Fig. 3; Table 4) vs 72cpgs (Fig. 4, Fig. S2), and a smaller increase with the barley-rooted, than the rye-rooted, inferences. I do not have plausible explanations for such increases of age estimation yet.

Some sequence heterogeneity was detected among 22 published circular cp genomes (Table S1). Such heterogeneity may have reflected the inversion heterogeneity^65,66^, the complex cpDNA structures^67^, or even the poor quality of assembling or sequencing^28^, as the quality issue of cp genome sequences is not new^21,23,57,68^ and the quality of the first cp genome sequence of *T. aestivum* cultivar ‘Chinese Spring’ was questioned^68^. Also, the substantial variations in substitution observed among the samples of the same species or subspecies in 72cpgs might have included the sequencing and/or assembling errors, or could even suggest the presence of sample or subspecies identity issues, particularly in those samples of *T. aestivum* spp. *maca* and *T. turgidum* subspecies. As the exact causes for those variations remain unknown, however, the effects of sequencing and/or assembling errors cannot be ruled out on the analyses presented here, particularly for those phylogenetic inferences and lineage dating. It could be reasoned that the effects on lineage dating in the MCC trees of 16cpgs may be smaller than those of 78cpgs. Nevertheless, further research is needed on the heterogeneity or quality issues and their effects on the inferences of maternal divergence.

The comparative analyses of 16cpgs and 72cpgs (or 78cpgs) also generated some novel results, as such comparative analyses with respect to sample size have rarely been conducted before. The analyses of more cp genomes detected more SNPs (Table 2), more codons showing positive selection (Table 3) and fewer SSRs per sample (Table S4). Such analyses also influenced the estimation of branch length in the phylogenetic trees and inflated the lineage age estimation (Fig. 3A vs Fig. 4). However, the use of more cp genomes did not alter the cp genome-wide patterns of nucleotide diversity variation observed for the complex (Fig. 2) and showed little impacts on the inferred phylogenetic tree topologies of the complex (Fig. 3A vs Fig. 4). These results are useful for future cp genome characterizations and phylogenetic inferences.

## Concluding remarks

The de novo cp genome assembling generated five new circular and annotated chloroplast genome sequences. More than 80% SNPs detected resided on the downstream and upstream gene regions and only 2.78% or less SNPs were predicted to be deleterious. Relatively weak selection pressure on the chloroplast coding genes was detected. The phylogenetic analyses confirmed that the maternal divergence of the *Triticum–Aegilops* complex had three deep lineages each representing a diploid species with nuclear A, B, or D genome. The maternal divergence between the emmer and bread wheats occurred at 8200–11,200 years ago. These findings are useful for further genomic studies and are significant for understanding the chloroplast genome evolvability and maternal divergence of the *Triticum–Aegilops* complex.

## Supplementary Information

Supplementary Information 1.
