# Supplementary material for: Characterizing chloroplast genomes and inferring maternal divergence of the Triticum–Aegilops complex
Source: Sci Rep. 2021 Jul 28;11:15363. doi: 10.1038/s41598-021-94649-9 (PMC8319314; doi:10.1038/s41598-021-94649-9)

Scientific Reports (doi.org/10.1038/s41598-021-94649-9)

Characterizing chloroplast genomes and inferring maternal divergence of the *Triticum-Aegilops* complex

Yong-Bi Fu

Plant Gene Resources of Canada, Saskatoon Research and Development Centre, Agriculture and Agri-Food Canada, 107 Science Place, Saskatoon, SK S7N 0X2, Canada. Phone: 306-385-9298, Fax: 306-385- 9489, email: yong-bi.fu@agr.gc.ca

Supplementary Materials : The supplementary materials consist of five supplemental tables and two supplemental figures as below:

Table S1. List of 95 complete circular and six incomplete (in red color) chloroplast genome sequences published in the National Centre for Biotechnology Information (NCBI) database (including 16 generated in this paper) representing 11 *Aegilops* and six *Triticum* species**.**

Table S2. List of 16 samples representing five *Aegilops* and six *Triticum* species and the related information on inventory, sequencing, genome assembly and GenBank accession.

Table S3. List of 131 genes and their duplications in the plastids of 16 *Triticum-Aegilops* samples.

Table S4. Simple sequence repeat (SSR) polymorphism detected in the 72 plastids with published circular chloroplast genome sequences of the *Triticum-Aegilops* complex.

Table S5. Results of the codeml analyses for testing positive selections of individual codons in chloroplast genes in the 16 chloroplast genome assemblies generated in this study (the upper section) and in the published 72 circular chloroplast genome sequences (the lower section).

Figure S1. Gene maps of five major *Triticum* and *Aegilops* chloroplast genomes: (A) *T. urartu* with nuclear genome Au; (B) *Ae. speltoides* with genome B; (C) *T. turgidum* ssp. *durum* with genome AuB; (D) *Ae. tauschii* with genome D; and *T. aestivum* with genome AuBD.

Figure S2. Bayesian maximum clade credibility trees of published 72 complete and six incomplete chloroplast genomes representing six *Triticum* and 11 *Aegilops* species with nodal support (in posterior probability) and outgroup of *Secale cereale*, as inferred from chloroplast sequence variations using BEAST software.

Table S1. List of 95 complete circular and six incomplete (in red color) chloroplast genome sequences published in the National Centre for Biotechnology Information (NCBI) database (including 16 generated in this paper) representing 11 *Aegilops* and six *Triticum* species.

KJ614418.1	*Ae. bicornis*	1	Gornicki et al. 2014	AB042240.3	*T. aestivum*	*	Ogihara et al. 2002
MG958548	*Ae. comosa*		This paper	KJ592713.1	*T. aestivum*	26	Bahieldin et al. 2014
KU207226.1	*Ae. cylindrica*	2	Gogniashvili et al. 2016	MW648991.1	*T. aestivum*	27	Xu 2021 (unpublished)
KU207224.1	*Ae. cylindrica*	3	Gogniashvili et al. 2016	MG958554	*T. aestivum*		This paper
KJ614420.1	*Ae. kotschyi*	4	Gornicki et al. 2014	LC377169.1	*T. aestivum*	28	Gogniashvili et al. 2018
KJ614416.1	*Ae. longissima*	5	Gornicki et al. 2014	LC005978.1	*T. aestivum* ssp. *maca*	29	Gogniashvili et al. (unpublished)
MG958549	*Ae. longissima*		This paper	LC375773.1	*T. aestivum* ssp. *maca*	30	Gogniashvili et al. 2018
KJ614415.1	*Ae. searsii*	6	Gornicki et al. 2014	LC375536.1	*T. aestivum* ssp. *maca*	31	Gogniashvili et al. 2018
KJ614413.1	*Ae. searsii*	7	Gornicki et al. 2014	LC373211.1	*T. aestivum* ssp. *maca*	32	Gogniashvili et al. 2018
KJ614414.1	*Ae. searsii*	8	Gornicki et al. 2014	LC372826.1	*T. aestivum* ssp. *maca*	33	Gogniashvili et al. 2018
KJ614419.1	*Ae. sharonensis*	9	Gornicki et al. 2014	NC_025955.1	*T. aestivum* ssp. *maca*	34	Gogniashvili et al. 2018
KJ614417.1	*Ae. sharonensis*	10	Gornicki et al. 2014	LC374397.1	*T. aestivum* ssp. *maca*	35	Gogniashvili et al. 2018
MG958553	*Ae. speltoides*		This paper	MG958556	*T. aestivum* ssp. *spelta*		This paper
KJ614405.1	*Ae. speltoides* var. *ligustica*	11	Gornicki et al. 2014	KJ614403.1	*T. aestivum* ssp. *spelta*	36	Gornicki et al. 2014
KJ614404.1	*Ae. speltoides* var. *ligustica*	12	Gornicki et al. 2014	KX631429.1	*T. aestivum* ssp. *tibeticum*	37	Cheng 2018 (unpublished)
KJ614406.1	*Ae. speltoides* var. *speltoides*	13	Gornicki et al. 2014	MG958551	*T. monococcum* ssp. *aegilopoides*		This paper
KU207225.1	*Ae. tauschii*	14	Gogniashvili et al. 2016	MG958558	*T. monococcum* ssp. *monococcum*		This paper
KU207223.1	*Ae. tauschii*	15	Gogniashvili et al. 2016	LC005977.1	*T. monococcum* ssp. *monococcum*	38	Gogniashvili et al 2014
KU207222.1	*Ae. tauschii*	16	Gogniashvili et al. 2016	MG958559	*T. timopheevii* ssp. *armeniacum*		This paper
KU198486.1	*Ae. tauschii*	17	Gogniashvili et al. 2016	KJ614409.1	*T. timopheevii* ssp. *armeniacum*	39	Gornicki et al. 2014
KU198485.1	*Ae. tauschii*	18	Gogniashvili et al. 2016	KJ614408.1	*T. timopheevii* ssp. *armeniacum*	40	Gornicki et al. 2014
KU198484.1	*Ae. tauschii*	19	Gogniashvili et al. 2016	KJ614407.1	*T. timopheevii* ssp. *armeniacum*	41	Gornicki et al. 2014
KU198483.1	*Ae. tauschii*	20	Gogniashvili et al. 2016	MG958546	*T. timopheevii* ssp. *timopheevii*		This paper
KU198482.1	*Ae. tauschii*	21	Gogniashvili et al. 2016	AB976560.1	*T. timopheevii* ssp. *timopheevii*	42	Gogniashvili et al 2014
KU198481.1	*Ae. tauschii*	22	Gogniashvili et al. 2016	KJ614410.1	*T. timopheevii* ssp. *timopheevii*	43	Gornicki et al. 2014
KJ614412.1	*Ae. tauschii*	23	Gornicki et al. 2014	KJ614397.1	*T. turgidum* ssp. *carthlicum*	44	Gornicki et al. 2014
MN223978.1	*Ae. tauschii*	*	Su et al. 2020	KJ614399.1	*T. turgidum* ssp. *carthlicum*	45	Gornicki et al. 2014
MN223977.1	*Ae. tauschii*	*	Su et al. 2020	LC376795.1	*T. turgidum* ssp. *carthlicum*	46	Gogniashvili et al. 2018
MN223976.1	*Ae. tauschii*	*	Su et al. 2020	LC375535.1	*T. turgidum* ssp. *carthlicum*	47	Gogniashvili et al. 2018
MN223975.1	*Ae. tauschii*	*	Su et al. 2020	MG958552	*T. turgidum* ssp. *dicoccoides*		This paper
MN258090.1	*Ae. tauschii*	*	Su et al. 2020	KJ614400.1	*T. turgidum* ssp. *dicoccoides*	48	Gornicki et al. 2014
MN258089.1	*Ae. tauschii*	*	Su et al. 2020	KJ614401.1	*T. turgidum* ssp. *dicoccoides*	49	Gornicki et al. 2014
MN258088.1	*Ae. tauschii*	*	Su et al. 2020	KJ614402.1	*T. turgidum* ssp. *dicoccoides*	50	Gornicki et al. 2014
MN258087.1	*Ae. tauschii*	*	Su et al. 2020	MG958550	*T. turgidum* ssp. *dicoccum*		This paper
MN258086.1	*Ae. tauschii*	*	Su et al. 2020	MG958545	*T. turgidum* ssp. *durum*		This paper
MN258085.1	*Ae. tauschii*	*	Su et al. 2020	KM352501.1	*T. turgidum* ssp. *durum*	51	Nie et al 2014 (unpublished)
MN258084.1	*Ae. tauschii*	*	Su et al. 2020	LC377262.1	*T. turgidum* ssp. *durum*	52	Gogniashvili et al. 2018
MN258083.1	*Ae. tauschii*	*	Su et al. 2020	KJ614398.1	*T. turgidum* ssp. *durum*	53	Gornicki et al. 2014
MN258082.1	*Ae. tauschii*	*	Su et al. 2020	MK253668.1	*T. turgidum* ssp. *paleocolchicum*	*	Nie et al. 2019 (unpublished)
MN258081.1	*Ae. tauschii*	*	Su et al. 2020	LC375229.1	*T. turgidum* ssp. *paleocolchicum*	54	Gogniashvili et al. 2018
MN258080.1	*Ae. tauschii*	*	Su et al. 2020	KJ614411.1	*T. urartu*	55	Gornicki et al. 2014
MN258079.1	*Ae. tauschii*	*	Su et al. 2020	KJ174105.1	*T. urartu*	56	Liu et al 2017 (unpublished)
MN258078.1	*Ae. tauschii*	*	Su et al. 2020	MG958555	*T. urartu*		This paper
MG958544	*Ae. tauschii*		This paper	MG958557	*T. zhukovskyi*		This paper
MG958547	*Ae. umbellulata*		This paper				
MK348601.1	*T. aestivum*	*	Liu et al. 2019	KY636071	*Ae. mutica*	57	Bernhardt et al. 2017
MH051715.1	*T. aestivum*	24	IWGSC et al. 2018	KY636072	*Ae. mutica*	58	Bernhardt et al. 2017
KJ614396.1	*T. aestivum*	25	Gornicki et al. 2014	KY636073	*Ae. mutica*	59	Bernhardt et al. 2017
CM022232.1	*T. aestivum*	**	Zimin et al. 2017	KY636074	*Ae. mutica*	60	Bernhardt et al. 2017
MK348611.1	*T. aestivum*	*	Liu et al. 2019	KY636075	*Ae. mutica*	61	Bernhardt et al. 2017
MK348610.1	*T. aestivum*	*	Liu et al. 2019	KY636076	*Ae. mutica*	62	Bernhardt et al. 2017

Note: The samples published by others have extra labels from 1 to 62 to distinguish among them and also from those assembled from this study. One star stands for poor quality (in sequencing, assembling or other unknown aspects) detected for a published genome sequence and two stars indicate no gene annotation; thus, they were excluded from the analysis in this study. Eleven related references are: Bernhardt et al. 2017 (10.1186/s12862-017-0989-9); Bahieldin et al. 2014 (10.1016/j.crvi.2014.07.001); Gogniashvili et al 2014 (10.1007/s10722-015-0230-x); Gogniashvili et al. 2016 (10.1007/s00294-016-0583-5) ; Gogniashvili et al. 2018 (10.1007/s10722-018-0671-0); Gornicki et al. 2014 (10.1111/nph.12931); IWGSC et al. 2018 (10.1126/science.aar7191); Liu et al. 2019 (10.1038/s41598-019-52786-2); Ogihara et al. 2002 (10.1007/s00438-001-0606-9); Su et al. 2020 (10.7717/peerj.8678) ; and Zimin et al. 2017 (10.1093/gigascience/gix097).

Table S2. List of 16 samples representing five *Aegilops* and six *Triticum* species and the related information on inventory, sequencing, genome assembly and GenBank accession.

Species	PGRC Acc#	Description	Origin/*donated* Country	Raw Reads	Coverage	Contig count	Scaffold count	CPG Size (bp)	GC%	NCBI Acc#	NCBI SRA Acc#
Ae. comosa	CN034253	WM	Greece	5232392	20015.9	17	5	136247	38.27	MG958548	SRR6705496
Ae. longissima	CN108024	WM	Israel	4281785	16558.0	5	3	136762	38.27	MG958549	SRR6705491
Ae. speltoides	CN108018	WM	Israel	2474394	10155.0	4	3	135982	38.27	MG958553	SRR6705494
Ae. tauschii	CN034229	WM	Iran	4562832	16937.8	5	3	135502	38.30	MG958544	SRR6705497
Ae. umbellulata	CN108095	WM	Italy	3667691	14125.0	5	3	136743	38.26	MG958547	SRR6705490
T. aestivum	CN011189	Neepawa	Canada	6308637	21467.8	5	3	135766	38.30	MG958554	SRR6705502
T. aestivum ssp. *spelta*	CN012261	BM	*Czech Republic*	4357654	14822.7	3	3	135819	38.29	MG958556	SRR6705500
T. monococcum ssp. *aegilopoides*	CN034176	WM	Iran	4831272	17566.9	5	3	136820	38.25	MG958551	SRR6705498
T. monococcum ssp. *monococcum*	CN052948	BM	Russia	4051857	14428.4	5	3	136758	38.25	MG958558	SRR6705492
T. timopheevii ssp. *armeniacum*	CN034138	WM	Turkey	4983128	18068.5	5	3	136757	38.25	MG958559	SRR6705499
T. timopheevii ssp. *timopheevii*	CN001847	BM	*Russia*	3032304	10372.9	5	3	136028	38.27	MG958546	SRR6705503
T. turgidum ssp. *dicoccoides*	CN107962	WM	Turkey	2962369	11521.1	3	3	135786	38.29	MG958552	SRR6705495
T. turgidum ssp. *dicoccum*	CN011272	BM	*Canada*	7544061	24003.7	5	3	135775	38.30	MG958550	SRR6705501
T. turgidum ssp. *durum*	CN041178	Leeds	USA	5275753	19683.0	5	3	135772	38.30	MG958545	SRR6705493
T. urartu	CN038614	WM	*Lebanon*	3990979	15276.2	4	3	136749	38.25	MG958555	SRR6705504
T. zhukovskyi	CN036212	WM	*Russia*	1698729	6417.1	4	3	136028	38.27	MG958557	SRR6705505

Note: PGRC=Plant Gene Resources of Canada; Acc#=accession number; WM=wild material; BM=breeding material; Neepawa and Leeds are two cultivars; donated country is shown in italics; CPG=chloroplast genome; SRA=Sequence Read Archive; and NCBI=National Centre for Biotechnology Information.

Table S3. List of 131 genes and their duplications in the plastids of 16 *Triticum-Aegilops* samples.

Category	Gene
Subunits of photosystem I	*psa*A, *psa*B, *psa*C, *psa*I, *psa*J
Subunits of photosystem II	*psb*A, *psb*B, *psb*C, *psb*D, *psb*E, *psb*F, *psb*H, *psb*I, *psb*J, *psb*K, *psb*L, *psb*M, *psb*T, *psb*Z
Subunits of cytochrome b/f complex	*pet*A, *pet*B[a], *pet*D[a], *pet*G, *pet*L, *pet*N
Subunits of ATP synthase	*atp*A, *atp*B, *atp*E, *atp*F[a], *atp*H, *atp*I
Large subunit of rubisco	*rbc*L
Subunits of NADH-dehydrogenase	*ndh*A[a], *ndh*B(1)[a], *ndh*C, *ndh*D, *ndh*E, *ndh*F, *ndh*G, *ndh*H, *ndh*I, *ndh*J, *ndh*K
Proteins of large ribosomal subunit	*rpl*2(1)[a], *rpl*14, *rpl*16[a], *rpl*20[a], *rpl*22, *rpl*23(2), *rpl*32, *rpl*33, *rpl*36
Proteins of small ribosomal subunit	*rps*2, *rps*3, *rps*4, *rps*7(1), *rps*8, *rps*11, *rps*12(1)[a], *rps*14, *rps*15(1), *rps*16[a], *rps*18, *rps*19(1)
Subunits of RNA polymerase	*rpo*A, *rpo*B, *rpo*C1, *rpo*C2
Cytochrome c biogenesis	*ccs*A
Transfer RNAs	*trn*A(1)[a], *trn*R(2), *trn*N(1), *trn*D, *trn*C, *trn*Q, *trn*E, *trn*G(1)[a], *trn*H(1), *trn*I(3)[a], *trn*L(3), *trn*K[a], *trn*M, *trnf*M(1), *trn*F, *trn*P, *trn*S(2), *trn*T(1), *trn*W, *trn*Y, *trn*V(2)[a]
Ribosomal RNAs	*16S rRNA*(1), *23S rRNA*(1), *4.5S rRNA*(1), *5S rRNA*(1)
Maturase	*mat*K
Protease	*clp*P1*
Photosystem assembly factors	*paf*I[a]*, *paf*II*, *pbf*1*
Envelope membrane protein	*cem*A
Translation initiation factor	*inf*A

Note: The superscript [a] indicates the gene transcript was joined and/or contains introns. The number in parentheses after a gene indicates the number of additional copies of the gene in the genome. * indicates revision of gene names from *psb*N to *pbf*1 (Krech et al. 2013 Plant Journal 75:1062-1074), *clp*P to *clp*P1 (Adam et al. 2006 Curr Opin Plant Biol 9:234-240), or *ycf*3 to *paf*I and *ycf*4 to *paf*II (Wicke et al. 2011 Plant Mol Biol 76:273-297).

Table S4. Simple sequence repeat (SSR) polymorphism detected in the 72 plastids with published circular chloroplast genome sequences of the *Triticum-Aegilops* complex.

SSR motif		A/T													C/G		AT/TA	AAT/ATA	
Repeat		10	11	12	13	14	15	16	17	18	19	21	22	26	10	11	6	5	Total
16 cp in this study																			
MG958548	*Ae. comosa*	10	11	4	2	0	0	0	0	1	0	0	0	0	2	0	0	1	31
MG958549	*Ae. longissima*	12	4	4	2	0	1	1	0	0	0	0	0	0	2	0	0	1	27
MG958553	*Ae. speltoides*	9	2	5	1	2	1	1	0	1	0	0	0	0	2	0	1	1	26
MG958544	*Ae. tauschii*	10	7	2	0	2	0	0	0	0	0	0	0	0	0	0	1	1	23
MG958547	*Ae. umbellulata*	13	6	1	1	1	1	0	0	0	0	0	0	0	2	0	1	1	27
MG958554	*T. aestivum*	9	4	4	1	3	2	1	0	0	0	0	0	0	1	0	1	1	27
MG958556	*T. aestivum* ssp. *spelta*	6	8	4	3	0	0	0	1	0	0	0	0	0	1	0	1	1	25
MG958551	*T. monococcum* ssp. *aegilopoides*	14	7	3	5	1	0	1	0	0	0	0	0	0	2	0	0	1	34
MG958558	*T. monococcum* ssp. *monococcum*	14	8	5	2	1	1	1	0	0	0	0	0	0	2	0	0	1	35
MG958559	*T. timopheevii* ssp. *armeniacum*	10	10	4	5	0	0	1	0	0	0	0	0	0	2	0	0	1	33
MG958546	*T. timopheevii* ssp. *timopheevii*	12	4	5	1	0	0	0	1	1	0	0	0	0	2	0	1	1	28
MG958552	*T. turgidum* ssp. *dicoccoides*	7	5	2	4	2	1	2	0	0	0	0	0	0	0	0	1	1	25
MG958550	*T. turgidum* ssp. *dicoccum*	8	3	3	2	3	2	1	0	0	0	0	0	0	3	0	1	1	27
MG958545	*T. turgidum* ssp. *durum*	9	4	4	1	2	3	0	1	0	0	0	0	0	3	0	1	1	29
MG958555	*T. urartu*	12	6	5	4	2	1	0	0	0	0	0	0	0	2	0	0	1	33
MG958557	*T. zhukovskyi*	12	5	5	1	0	0	0	1	1	0	0	0	0	2	0	1	1	29
Published 56 cp																			
KJ614418.1	*Ae. bicornis*	10	7	2	0	0	0	2	0	0	0	0	0	0	0	0	0	1	22
KU207226.1	*Ae. cylindrica*	8	8	2	1	0	0	0	0	0	0	0	0	0	0	0	1	1	21
KU207224.1	*Ae. cylindrica*	8	8	3	1	0	0	0	0	0	0	0	0	0	0	0	1	1	22
KJ614420.1	*Ae. kotschyi*	13	7	2	0	0	0	2	0	0	0	0	0	0	0	0	0	1	25
KJ614416.1	*Ae. longissima*	12	5	2	0	0	0	1	0	1	0	0	0	0	0	0	0	1	22
KJ614415.1	*Ae. searsii*	13	6	2	0	0	0	2	0	0	0	0	0	0	0	0	0	1	24
KJ614413.1	*Ae. searsii*	12	5	2	0	0	0	1	0	1	0	0	0	0	0	0	0	1	22
KJ614414.1	*Ae. searsii*	13	7	2	0	0	0	2	0	0	0	0	0	0	0	0	0	1	25
KJ614419.1	*Ae. sharonensis*	13	4	2	0	0	0	2	0	0	0	0	0	0	0	0	0	1	22
KJ614417.1	*Ae. sharonensis*	13	5	2	0	0	0	2	0	0	0	0	0	0	0	0	0	1	23
KJ614405.1	*Ae. speltoides* var. *ligustica*	9	4	4	2	0	0	0	1	1	0	0	0	0	2	0	1	1	25
KJ614404.1	*Ae. speltoides* var. *ligustica*	9	4	4	2	0	0	0	1	1	0	0	0	0	2	0	1	1	25
KJ614406.1	*Ae. speltoides* var. *speltoides*	8	4	4	2	0	0	0	1	1	0	0	0	0	2	0	1	1	24
KJ614412.1	*Ae. tauschii*	13	8	3	0	0	0	0	0	0	0	0	0	0	0	0	1	1	26
KU207225.1	*Ae. tauschii*	12	5	4	0	0	0	0	0	0	0	0	0	0	0	1	0	1	23
KU207223.1	*Ae. tauschii*	8	9	2	1	0	0	0	0	0	0	0	0	0	0	0	1	1	22
KU207222.1	*Ae. tauschii*	12	4	3	0	0	0	0	0	0	0	0	0	0	0	1	0	1	21
KU198486.1	*Ae. tauschii*	6	10	2	1	0	0	0	0	0	0	0	0	0	0	0	0	1	20
KU198485.1	*Ae. tauschii*	8	6	3	5	0	0	0	0	0	0	0	0	0	0	0	1	1	24
KU198484.1	*Ae. tauschii*	8	9	2	0	0	0	0	0	0	0	0	0	0	0	0	1	1	21
KU198483.1	*Ae. tauschii*	11	6	3	0	0	0	0	0	0	0	0	0	0	0	0	1	1	22
KU198482.1	*Ae. tauschii*	6	10	2	1	0	0	0	0	0	0	0	0	0	0	0	0	1	20
KU198481.1	*Ae. tauschii*	9	8	2	1	0	0	0	0	0	0	0	0	0	0	0	1	1	22
LC377169.1	*T. aestivum*	7	6	4	1	3	2	1	0	0	0	0	0	0	1	0	1	1	27
KJ614396.1	*T. aestivum*	10	4	4	0	4	2	0	1	0	0	0	0	0	1	0	1	1	28
MH051715.1	*T. aestivum*	9	4	4	1	3	1	0	1	0	0	1	0	0	1	0	1	1	27
KJ592713.1	*T. aestivum*	9	3	5	1	3	2	0	1	0	0	0	0	0	1	0	1	1	27
MW648991.1	*T. aestivum*	9	3	5	1	3	2	0	1	0	0	0	0	0	1	0	1	1	27
LC374397.1	*T. aestivum* ssp. *maca*	9	5	2	2	4	1	1	0	0	0	0	0	0	1	0	1	1	27
LC005978.1	*T. aestivum* ssp. *maca*	8	5	4	1	3	2	1	0	0	0	0	0	0	1	0	1	1	27
NC_025955.1	*T. aestivum* ssp. *maca*	8	5	4	1	3	2	1	0	0	0	0	0	0	1	0	1	1	27
LC372826.1	*T. aestivum* ssp. *maca*	9	4	3	2	3	2	1	0	0	0	0	0	0	1	0	1	1	27
LC373211.1	*T. aestivum* ssp. *maca*	9	4	3	2	3	2	1	0	0	0	0	0	0	1	0	1	1	27
LC375536.1	*T. aestivum* ssp. *maca*	9	4	3	2	3	2	1	0	0	0	0	0	0	1	0	1	1	27
LC375773.1	*T. aestivum* ssp. *maca*	9	4	3	2	3	2	1	0	0	0	0	0	0	1	0	1	1	27
KJ614403.1	*T. aestivum* ssp. *spelta*	9	5	5	0	3	0	1	1	0	0	0	0	0	1	0	1	1	27
KX631429.1	*T. aestivum* ssp. *tibeticum*	9	4	4	1	3	2	1	0	0	0	0	0	0	1	0	1	1	27
LC005977.1	*T. monococcum* ssp. *monococcum*	12	7	7	2	1	1	0	0	0	0	0	0	0	0	0	0	1	31
KJ614409.1	*T. timopheevii* ssp. *armeniacum*	11	5	5	1	0	0	0	1	1	0	0	0	0	2	0	1	1	28
KJ614408.1	*T. timopheevii* ssp. *armeniacum*	11	5	5	1	0	0	0	1	1	0	0	0	0	2	0	1	1	28
KJ614407.1	*T. timopheevii* ssp. *armeniacum*	10	5	5	1	0	0	0	1	1	0	0	0	0	2	0	1	1	27
AB976560.1	*T. timopheevii* ssp. *timopheevii*	12	4	5	1	0	0	1	0	1	0	0	0	0	2	0	1	1	28
KJ614410.1	*T. timopheevii* ssp. *timopheevii*	11	5	5	1	0	0	0	1	1	0	0	0	0	2	0	1	1	28
KJ614397.1	*T. turgidum* ssp. *carthlicum*	10	4	4	0	4	2	0	1	0	0	0	0	0	1	0	1	1	28
KJ614399.1	*T. turgidum* ssp. *carthlicum*	10	3	4	0	5	2	0	1	0	0	0	0	0	1	0	1	1	28
LC375535.1	*T. turgidum* ssp. *carthlicum*	9	4	4	1	3	3	0	0	0	0	0	0	0	1	0	1	1	27
LC376795.1	*T. turgidum* ssp. *carthlicum*	9	4	4	1	3	2	1	0	0	0	0	0	0	1	0	1	1	27
KJ614400.1	*T. turgidum* ssp. *dicoccoides*	11	4	4	0	4	2	0	1	0	0	0	0	0	1	0	1	1	29
KJ614401.1	*T. turgidum* ssp. *dicoccoides*	9	5	4	0	4	1	0	1	0	0	0	0	0	0	0	1	1	26
KJ614402.1	*T. turgidum* ssp. *dicoccoides*	9	5	3	0	3	1	1	1	0	0	0	0	0	0	0	1	1	25
KM352501.1	*T. turgidum* ssp. *durum*	9	4	1	2	0	1	1	1	0	1	0	1	1	1	0	1	1	25
LC377262.1	*T. turgidum* ssp. *durum*	9	5	3	1	3	2	0	1	0	0	0	0	0	1	0	1	1	27
KJ614398.1	*T. turgidum* ssp. *durum*	10	4	4	0	4	2	0	1	0	0	0	0	0	1	0	1	1	28
LC375229.1	*T. turgidum* ssp. *paleocolchicum*	9	4	4	1	3	2	1	0	0	0	0	0	0	1	0	1	1	27
KJ614411.1	*T. urartu*	14	4	4	0	1	0	0	1	1	0	0	0	0	0	0	0	1	26
KJ174105.1	*T. urartu*	9	4	4	1	2	3	0	1	0	0	0	0	0	1	0	1	1	27
Total		718	388	251	83	103	61	38	26	15	1	1	1	1	68	2	52	72	1881

Table S5. Results of the codeml analyses for testing positive selections of individual codons in chloroplast genes in the 16 chloroplast genome assemblies generated in this study (the upper section) and in the published 72 circular chloroplast genome sequences (the lower section). Log-likelihood values (InL) and parameter estimates under models of variable ω ratios among sites are shown.

Model code	lnL	P-value for LRT	Estimates of parameters	Count of PSS NEB	Count of PSS BEB
16 cp samples					
M0 (One-ratio)	-66615.55		ω=0.45118		
M3 (Discrete)	-66501.64	<0.000001	p0=0.84264, p1=0.14714, p2=0.01021		
			ω0=0.00000, ω1=0.00000, ω2=22.24656		
M1a (Nearly neutral)	-66570.06		p0=0.88735, (p1=0.11265), ω0=0.00000, (ω1=1.00000)		
M2a (Positive selection)	-66553.45	<0.000001	p0=0.94103, p1=0.00107, (p2=0.05790),	81(81)	3(0)
			ω0=0.00000, (ω1=1.00000), ω2=2.00000		
M7 (Beta)	-66584.04		p=0.12298, q=0.62025		
M8 (Beta&ω)	-66480.88	<0.000001	p0=0.99410, p=0.00500, q=99.00000, (p1=0.00590), ω=999.00000	81(81)	30(29)
M8a (Beta&ω>1)	-66570.39		p0=0.89180, p=0.00500, q=1.03068, (p1=0.10820), ω=1.00000		
M8 (Beta&ω)	-66480.88	<0.000001	p0=0.99410, p=0.00500, q=99.00000, (p1=0.00590), ω=999.00000	81(81)	30(29)
72 cp samples					
M0 (One-ratio)	-97493.51		ω=0.40199		
M3 (Discrete)	-55095.26	<0.000001	p0=0.82700, p1=0.15373, p2=0.01927		
			ω0=0.00000, ω1=0.02759, ω2=140.87994		
M1a (Nearly neutral)	-55425.80		p0=0.91979, (p1=0.08021), ω0=0.00000, (ω1=1.00000)		
M2a (Positive selection)	-55333.04	<0.000001	p0=0.94946, p1=0.00000, (p2=0.05054),	243(243)	44(5)
			ω0=0.00000, (ω1=1.00000), ω2=2.00000		
M7 (Beta)	-55435.61		p=0.00741, q=0.05820		
M8 (Beta&ω)	-55095.16	<0.000001	p0=0.98092, p=0.00774, q=0.25854, (p1=0.01908), ω=168.40765	243(243)	141(135)
M8a (Beta&ω>1)	-55424.26		p0=0.91750, p=0.00500, q=1.51681, (p1=0.08250), ω=1.00000		
M8 (Beta&ω)	-55095.16	<0.000001	p0=0.98092, p=0.00774, q=0.25854, (p1=0.01908), ω=168.40765	243(243)	141(135)

Note: LRT = likelihood ration test; PSS = positively selected site; NEB = Naïve Empirical Byes analysis; BEB = Bayesian Empirical Bayes analysis; and the first number is the count of PSS with posterior probabilities >95%, and the second number (in parenthesis) is the count of PSS with posterior probabilities >99%.

Figure S1. Gene maps of five major *Triticum* and *Aegilops* chloroplast genomes: (A) *T. urartu* with nuclear genome Au; (B) *Ae. speltoides* with genome B; (C) *T. turgidum* ssp. *durum* with genome AuB; (D) *Ae. tauschii* with genome D; and *T. aestivum* with genome AuBD. Each map is represented in, moving counterclockwise from the right. The larger circle represents the layout of chloroplast genes distribution as per their transcription direction: outside boxes and inside boxes show the counterclockwise and clockwise transcription. The colour of the gene box indicates the functional group that the gene belongs to. The smaller circle represents the CG content plot in the corresponding sample. LSC, large single copy region; SSC, small single copy region. IRa/b, inverted repeats. Intron-containing genes are marked by a '∗' symbol and pseudogenes are marked by a 'Ψ' symbol. Note that the maps were generated using GenomeVx[40] (http://wolfe.ucd.ie/GenomeVx/) and Circos version 0.69-4[41] (http://www.circos.ca/software/) and merged in Inkscape 0.92 (https://inkscape.org).

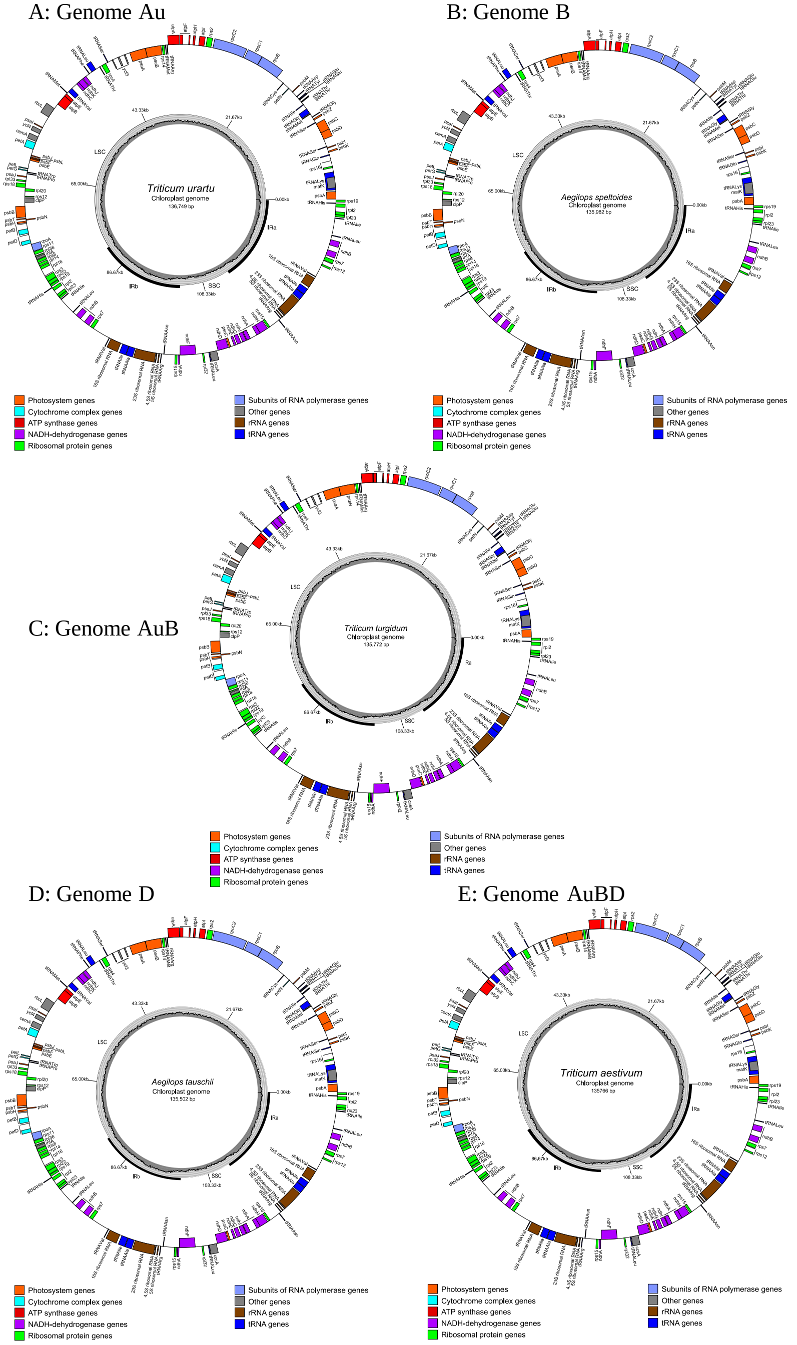

Figure S2. Bayesian maximum clade credibility trees of the published 72 complete and six incomplete chloroplast genomes representing six *Triticum* and 11 *Aegilops* species with nodal support (in posterior probability) and outgroup of *Secale cereale*, as inferred from chloroplast sequence variations using BEAST software. A sample with a number after its species name was published by others and a sample without the number label was assembled from this study (see Table S1). The nodes with a posterior probability of 0.90 or less are highlighted in red. The node ages were calibrated with the root ages in Mya following the related age estimates by Chalupska *et al.* (2008). Some lineage ages are shown, along the divergence time axis at the bottom of the figure. The trees shared the same topology as those inferred from the 16 chloroplast genomes generated in this study (Fig. 3) and the published 78 chloroplast genomes (Fig. 4), but carried many nodes with poor support toward hexaploid wheat. Also, the age estimates at the deep lineages appear to be increased when compared with those in Fig. 3.

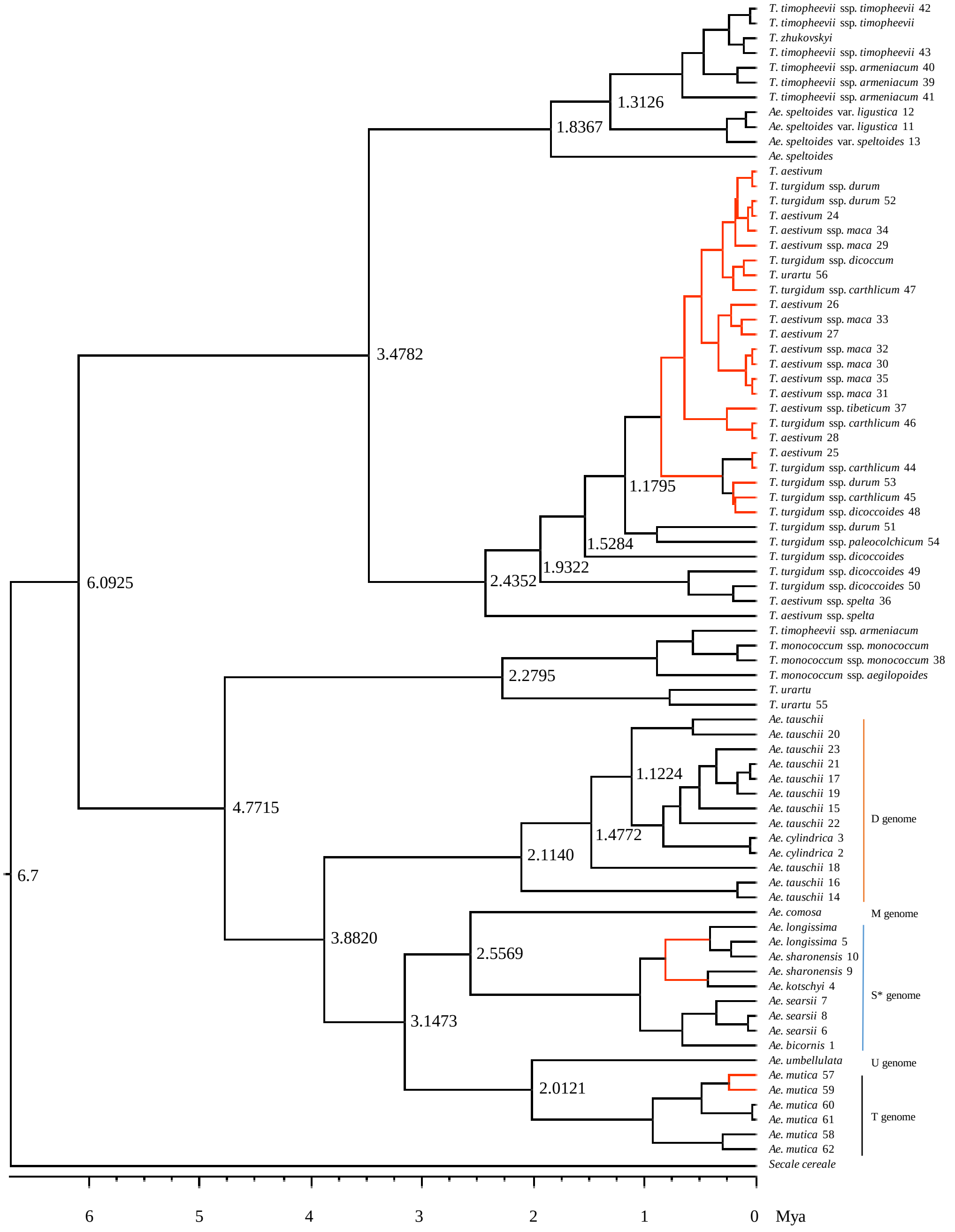

Supplement: Supplementary file 1 — Supplementary Information 1. [file 41598_2021_94649_MOESM1_ESM.pdf]
